# A Survey on Anticoagulation Practices in Patients with Chronic Kidney Disease in Saudi Arabia

**DOI:** 10.3390/jcm15176517

**Published:** 2026-08-23

**Authors:** Mansoor Radwi, Enad Alsolami

**Affiliations:** Department of Internal Medicine, College of Medicine, University of Jeddah, Jeddah 23218, Saudi Arabia; eaalsolami@uj.edu.sa

**Keywords:** chronic kidney disease, anticoagulation, venous thrombosis, atrial fibrillation

## Abstract

**Background/Objectives**: Anticoagulation in advanced chronic kidney disease (CKD) remains challenging due to limited representation in clinical trials, resulting in uncertainty regarding optimal drug selection, dosing, and monitoring strategies. **Methods**: We conducted a cross-sectional, web-based survey of clinicians involved in CKD care and anticoagulation prescribing. The questionnaire explored respondent characteristics, methods of renal function estimation, anticoagulant selection across common CKD scenarios, anticoagulants’ dosing and monitoring practices, and approaches to anticoagulation in nephrotic syndrome. **Results**: Fifty-seven clinicians participated in the survey. Unfractionated heparin was the preferred agent for venous thromboembolism (VTE) prophylaxis in patients with stage 4 and 5 CKD, or on dialysis. For acute VTE treatment, anticoagulant choice varied by CKD severity, with unfractionated heparin, direct oral anticoagulants (DOACs), and low-molecular-weight heparin (LMWH) all reported to be used depending on stages of CKD. Warfarin and DOACs were commonly used for treatment of chronic VTE and atrial fibrillation (AF), with warfarin more frequently selected in patients receiving dialysis. Apixaban was the most frequently reported DOAC, typically at a dose of 2.5 mg twice daily in advanced CKD and dialysis. Renal function estimation methods varied, with Cockcroft–Gault most used overall, while nephrologists more frequently reported MDRD or CKD-EPI equations. Most respondents reported not routinely monitoring DOAC levels, and the majority indicated the absence of a structured multidisciplinary team (MDT) for managing anticoagulated CKD patients. Clinical practice varied according to specialty, experience, and healthcare setting, particularly in anticoagulant selection, dosing strategies, and use of risk stratification tools. **Conclusions**: Anticoagulation practices in advanced CKD are highly heterogeneous, reflecting persistent evidence gaps. While unfractionated heparin and warfarin remain central in patients with stage 5 CKD or on dialysis, DOAC use—particularly apixaban—has expanded, often with empiric dose reduction. These findings underscore the need for clearer guidance, standardized approaches to renal function assessment, multidisciplinary care pathways, and prospective studies focusing on anticoagulation use in CKD populations.

## 1. Introduction

Chronic kidney disease (CKD) is a significant health problem with an estimated prevalence of nearly 300 million globally [1]. The hemostatic profile in patients with CKD is complex. It is characterized by simultaneous increased susceptibility to thromboembolic events and hemorrhagic complications [2]. These dual opposite states are attributed to multiple factors, e.g., impaired platelet function, systemic vascular and metabolic dysfunction inherent to CKD. Hence, anticoagulation use is a challenge in such a population.

Indications for anticoagulation in CKD are primary and secondary prevention of atrial fibrillation-related stroke, venous thromboembolism, and, in select cases, the management of vascular access thrombosis in hemodialysis populations [3]. Traditional agents, such as vitamin K antagonist (VKAs), notably warfarin, and unfractionated or low-molecular-weight heparins (UFH/LMWH), have been the mainstay of therapy for decades. The advent of direct oral anticoagulants (DOACs) has transformed anticoagulation paradigms in the general population due to superior or comparable efficacy and predictability, thus obviating the need for routine monitoring required in patients on warfarin. However, use of DOACs in patients with moderate-to-severe CKD falls in an area of uncertainty [4]. Major randomized controlled trials (RCTs) typically excluded or underrepresented those with advanced renal impairment [5], creating gaps in high-quality evidence and thus creating uncertainty in clinical practice, as reflected in the variations of recommendations in major guidelines that discussed the use of anticoagulation in CKD [6].

Surveys carried out in various countries have revealed variation in practice as clinicians are compelled to balance incomplete evidence with the demands of individualized patient care [7,8]. Local data on anticoagulation practice in advanced CKD are limited; understanding clinician practice in Saudi Arabia may identify implementation gaps and help establish national or institutional anticoagulation pathways. The objective of this study is to: (1) assess current anticoagulation practices among clinicians managing CKD, (2) identify if certain factors, e.g., experience, may influence variation in practice, and (3) identify priority areas for educational and research interventions. 

## 2. Materials and Methods

We employed a cross-sectional, web-based survey targeting clinicians involved in the management of patients with CKD and who also prescribed anticoagulation for various reasons in this population. The survey was disseminated randomly through direct contact and through local hematology/nephrology groups. We targeted any active physicians who were classified by the Saudi Commission for Health Specialties (SCFHS) as a nephrologist or hematologist (consultant or specialist), or were training in a fellowship program in the field of hematology or nephrology at the time of filling the survey. Reminders to all participants were sent after 8 weeks following the initial invitation to enhance response rates.

The questionnaire used in this study was adapted from a previously published UK survey [8]. It consisted of both closed-ended (single- and multiple-choice) and hypothetical scenarios to capture quantitative and qualitative insights. The questions covered areas such as: (1) demographic and practice characteristics; (2) methods to assess renal functions prior to start anticoagulant; (3) hypothetical cases that assessed type anticoagulant used for venous thromboembolism (VTE) prophylaxis and dose, if applicable, in advance stages of CKD; (4) hypothetical cases that assessed type of anticoagulant used for treatment of VTE and dose, if applicable, in advance stages of CKD; (5) decision to anticoagulated in patients with advance stages of CKD according to risk stratification approaches (e.g., low or high risk of thrombosis according to CHA2DS2-VASc vs. low or high risk of bleeding according to HAS-BLED); (6) type and dose of anticoagulant in patients with advance stages of CKD with atrial fibrillation (AF); (7) use of anticoagulation in various severity of nephrotic syndrome. The results were presented as descriptive analysis for the cohort as a whole and according to experience, practice setting, and specialty.

Participation was voluntary and anonymous, with informed electronic consent obtained prior to survey initiation. No personally identifiable information was collected. Participants were allowed to withdraw their responses by contacting the authors through their emails.

## 3. Results

### 3.1. Respondent Characteristics

Survey responses were available from 57 clinicians. Two-thirds of respondents were male. Most respondents reported working in government hospitals (59.6%), followed by mixed government/private practice (19.3%), private hospitals (10.5%), and outpatient dialysis units only (10.5%). In terms of experience, 43.9% reported >10 years in practice, 26.3% reported 5–10 years, and 29.8% reported <5 years (see Table 1). The majority of responders were classified as consultants, and nearly two-thirds of them were in the nephrology track. Regarding training pathways, the most frequently selected route was Saudi Board training in hematology or nephrology (59.6%), followed by Canadian-based training (24.6%), USA-based training (10.5%), and UK-based training (8.8%) (see Table 1).

### 3.2. Type of Practice and Case-Mix

The most encountered type of patients in practice were patients receiving dialysis with acute VTE (87.7%), followed by patients receiving dialysis requiring medical VTE prophylaxis (80.7%), and patients receiving dialysis with AF (78.9%). Exposure to nephrotic syndrome (68.4%) and kidney transplant patients (57.9%) was lower. Physicians in the hematology track selected more frequently encountering patients on dialysis who developed acute VTE compared to their counterparts (95% vs. 84%), while physicians in the nephrology track selected more frequently encountering patients on dialysis with AF compared to their counterparts (89% vs. 60%). The latter also selected encountering more frequently patients who underwent kidney transplantation (65% vs. 45%) or nephrotic syndrome (75% vs. 55%) compared to their counterparts (see Table 1).

Nearly two-thirds of respondents reported the absence of a structured multidisciplinary team (MDT) for managing patients with CKD on anticoagulation, while one-fourth reported the presence of an MDT and almost one-tenth were unsure. Those who worked in governmental hospitals or outpatient dialysis more frequently selected the presence of an MDT compared to counterparts in private hospitals (see Table 1).

### 3.3. Questions Related to Cases of CKD

#### 3.3.1. Renal Function Estimation When Using Anticoagulation

**Figure 1:**
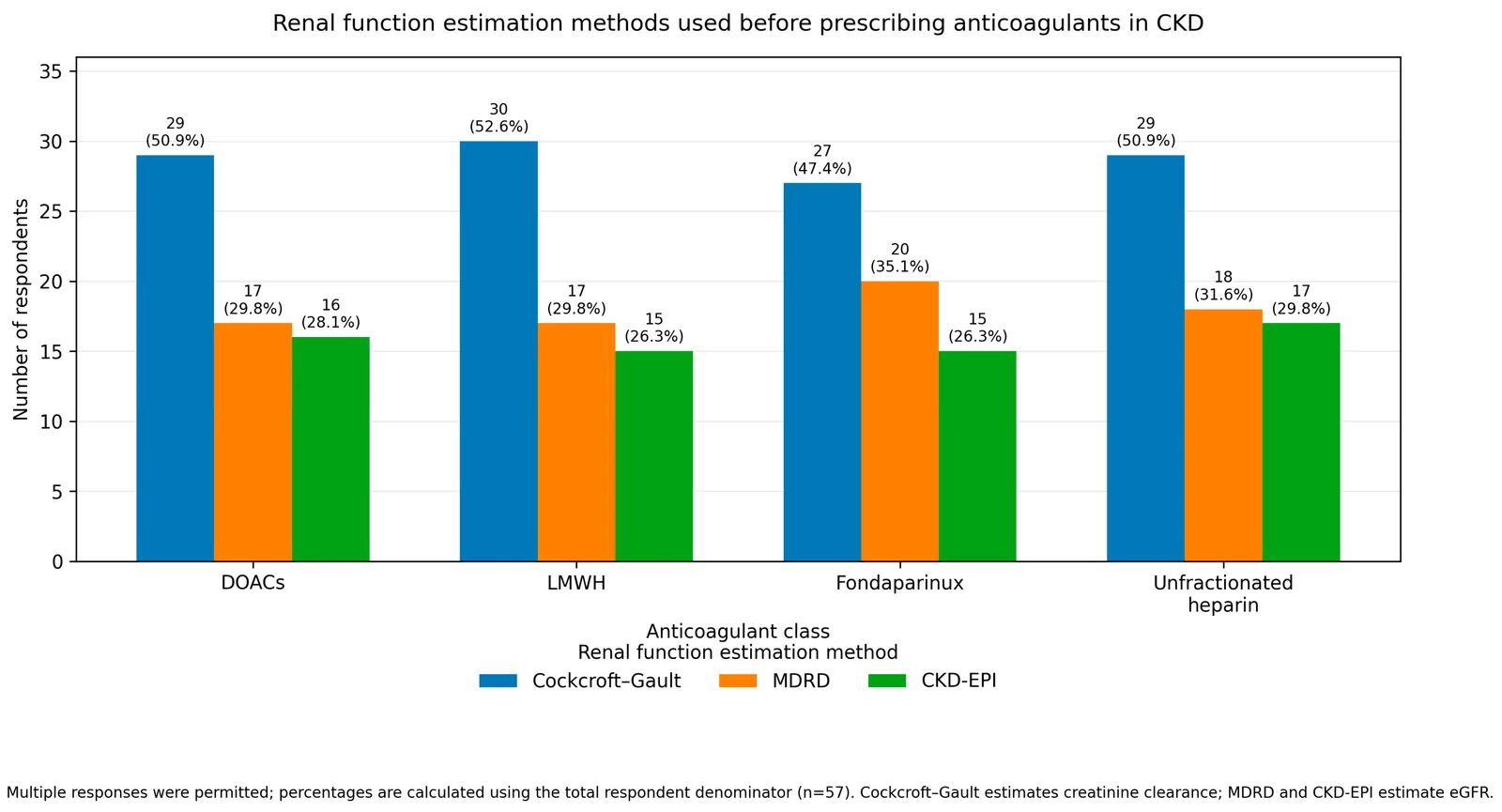
Assessment of renal function when using anticoagulants in CKD.

Across all anticoagulant classes, Cockcroft–Gault was the most frequently selected method for renal function estimation, followed by the Modification of Diet in Renal Disease (MDRD) method and CKD-EPI (see Figure 1).

***Deviation across subgroup analysis:*** variation was seen according to the type of healthcare system, where the majority of physicians working in governmental hospitals or those in outpatient dialysis units selected the Cockcroft–Gault method more frequently, while physicians working in private hospitals selected the MDRD method more frequently. Nephrologists selected more frequently CKD-EPI or MDRD while hematologists selected Cockcroft-Gault more frequently when prescribing any type of anticoagulation in patients with CKD (see Table S1 in Supplementary Materials).

#### 3.3.2. VTE Prophylaxis Choices and Alternatives

**Figure 2:**
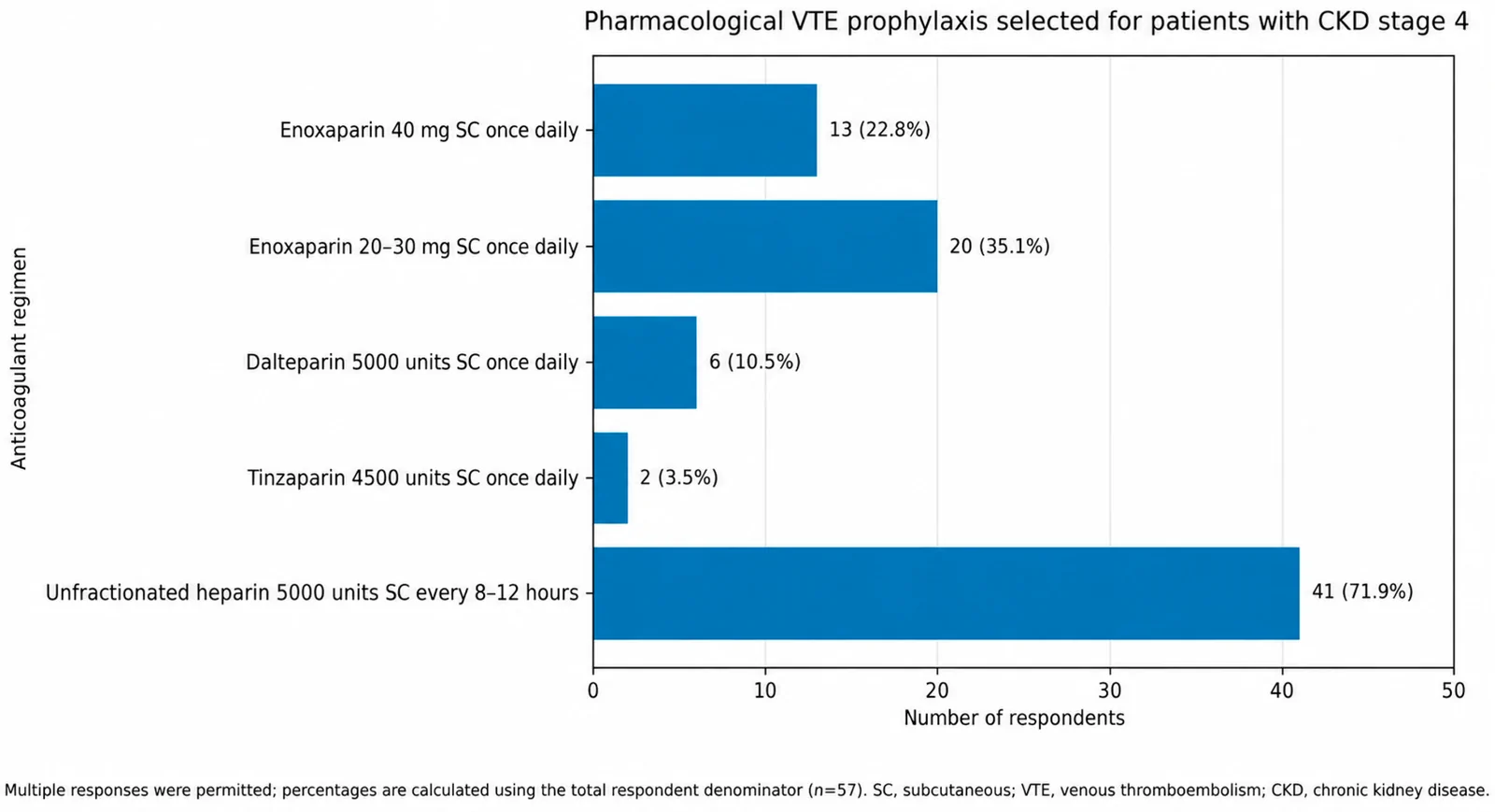
Type of pharmacological VTE PROPHYLAXIS used in patients with Stage 4 CKD (eGFR 15–29) (more than one option could be chosen).

**Figure 3:**
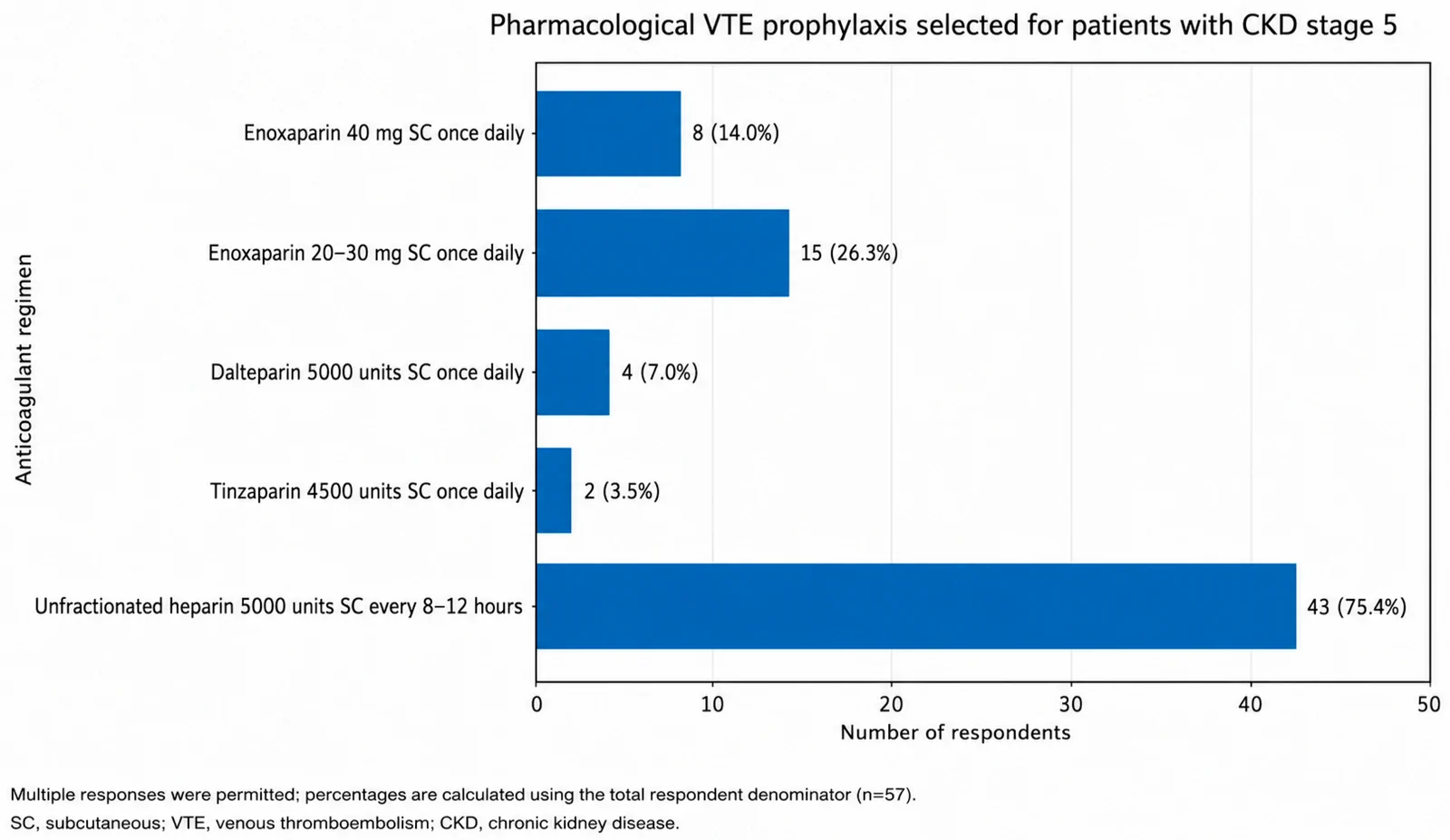
Type of pharmacological VTE PROPHYLAXIS used in patients with stage 5 (eGFR < 15) (more than one option could be chosen).

**Figure 4:**
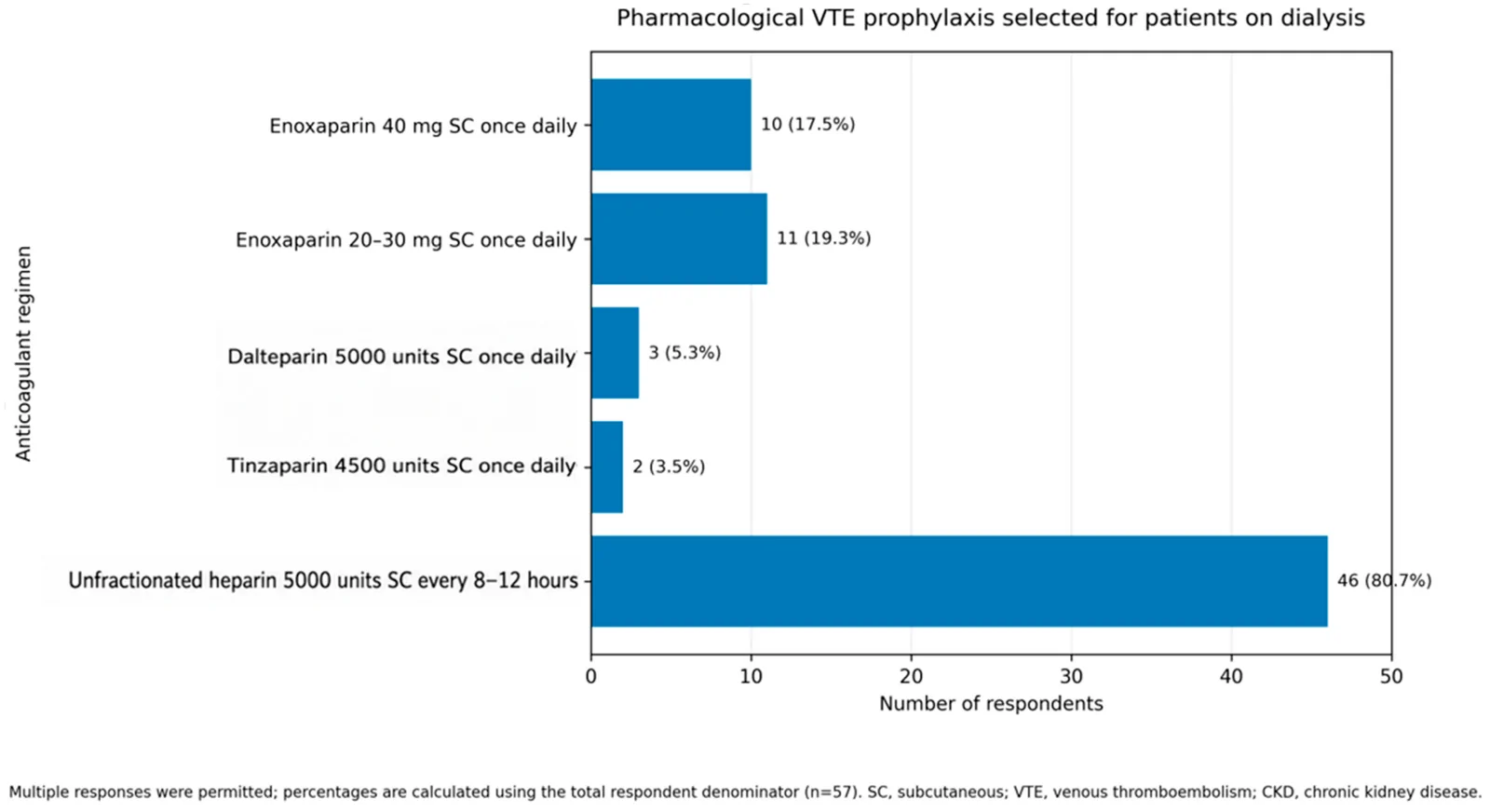
Type of pharmacological VTE prophylaxis used in patients on dialysis (more than one option could be chosen).

Type of pharmacological VTE prophylaxis used: unfractionated heparin (UFH) was selected most frequently for patients with stage 4 CKD (71.9%), stage 5 CKD (75.4%), or on dialysis (80.7%). Reduced-dose enoxaparin (20–30 mg daily) was selected by 35% of physicians for patients with stage 4 CKD, followed by 24.6% for patients with stage 5 CKD, and 19% on dialysis (see Figure 2, Figure 3 and Figure 4).

***Deviation across subgroup analysis:*** Physicians working in outpatient dialysis showed variability when selecting pharmacological VTE prophylaxis agents (i.e., equally selected enoxaparin and UFH) for patients with stage 4 and 5 CKD (see Table S2 in Supplementary Materials).

**Figure 5:**
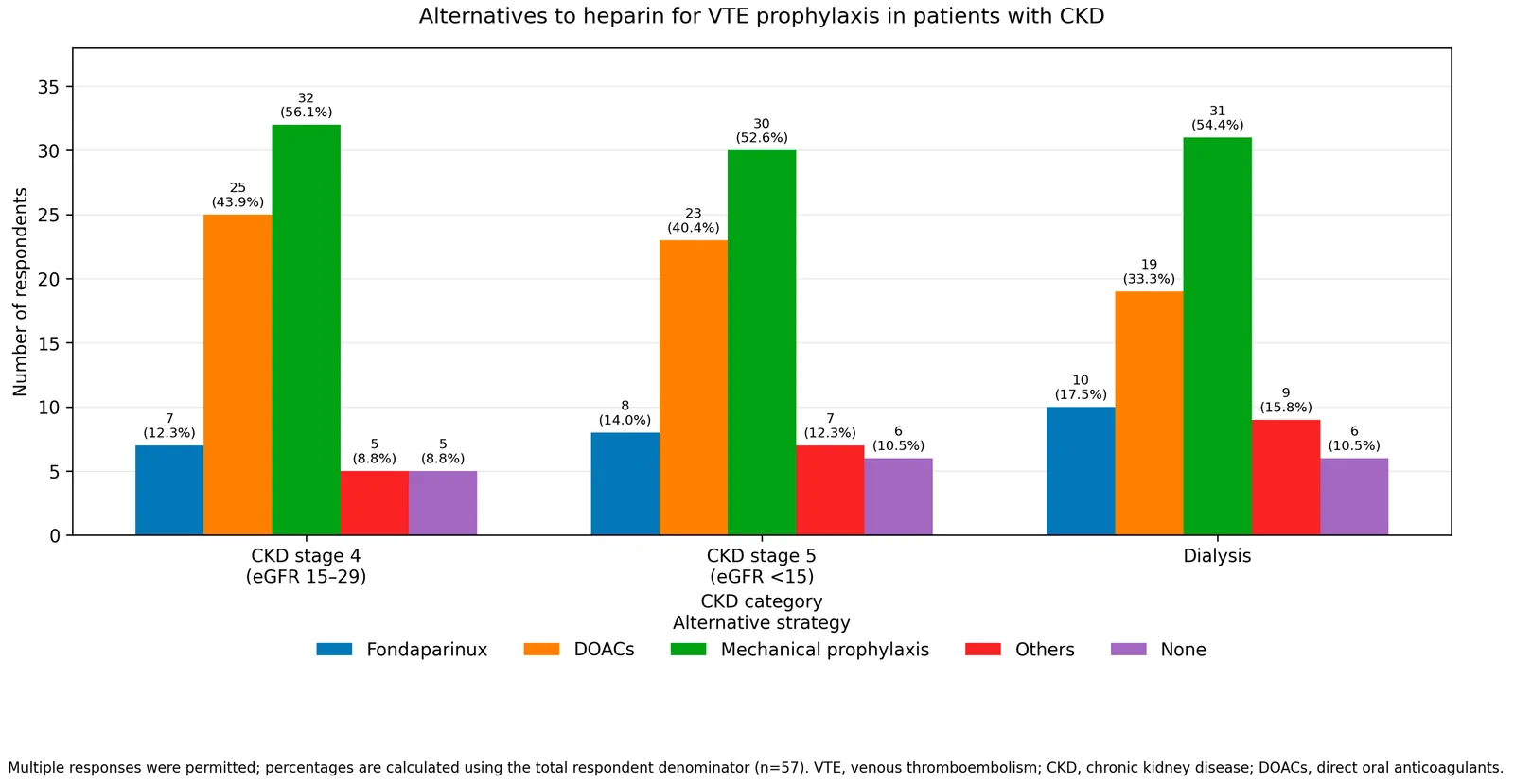
Anticoagulation agents alternative to heparin for VTE prophylaxis in patients with CKD.

Type of VTE prophylaxis used when heparin cannot be used: mechanical VTE prophylaxis method was the most frequently selected overall as an alternative to heparin (see Figure 5).

***Deviation across subgroup analysis:*** Physicians working in outpatient dialysis-only units selected DOACs more frequently as an alternative to heparin when choosing a VTE prophylaxis method for patients with advanced CKD, including dialysis, compared to their counterparts. Also, physicians with more than 10 years of experience selected DOACs more frequently as an alternative compared to counterparts (i.e., physicians who have less than 10 years of experience) (see Table S3 in Supplementary Materials).

#### 3.3.3. Acute and Chronic VTE Treatment

**Figure 6:**
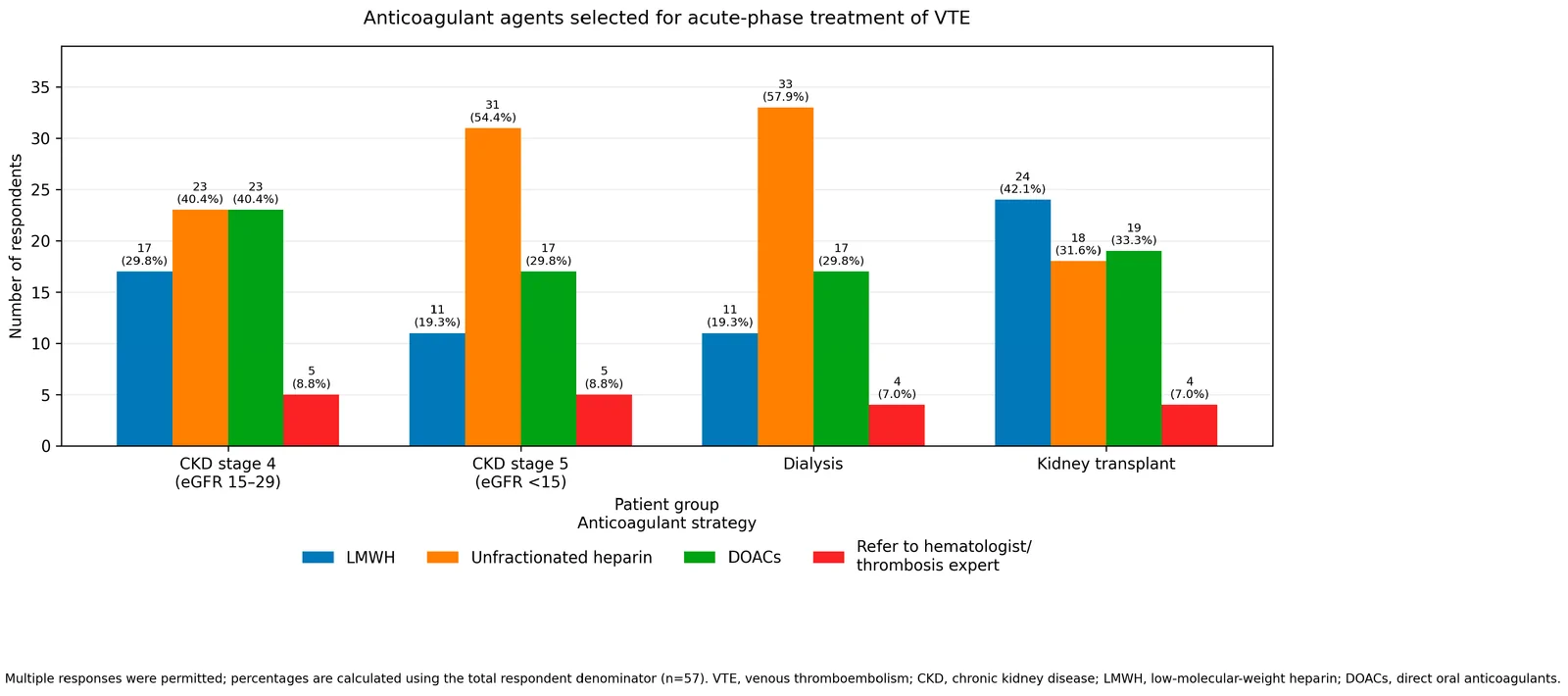
Anticoagulation agents used for acute phase treatment of VTE.

Treatment of acute VTE: UFH was selected most frequently, followed by DOACs for patients with stage 5 CKD or on dialysis. DOACs and UFH were both equally selected as the first option, followed by LMWH for patients with stage 4 CKD, while LMWH was selected more frequently, followed by DOACs or UFH, in patients with kidney transplants. Only a minority of respondents would refer them to hematologists or thrombosis experts (see Figure 6).

***Deviation across subgroup analysis:*** when compared to their counterparts, DOACs were more frequently selected by physicians working in private hospitals or in outpatient dialysis units for patients with stage 4 or 5 CKD. They were also selected more frequently by physicians working in private hospitals for patients with kidney transplants, and by physicians with less than 10 years of experience for patients with stage 4 CKD. (see Table S4 in Supplementary Materials).

**Figure 7:**
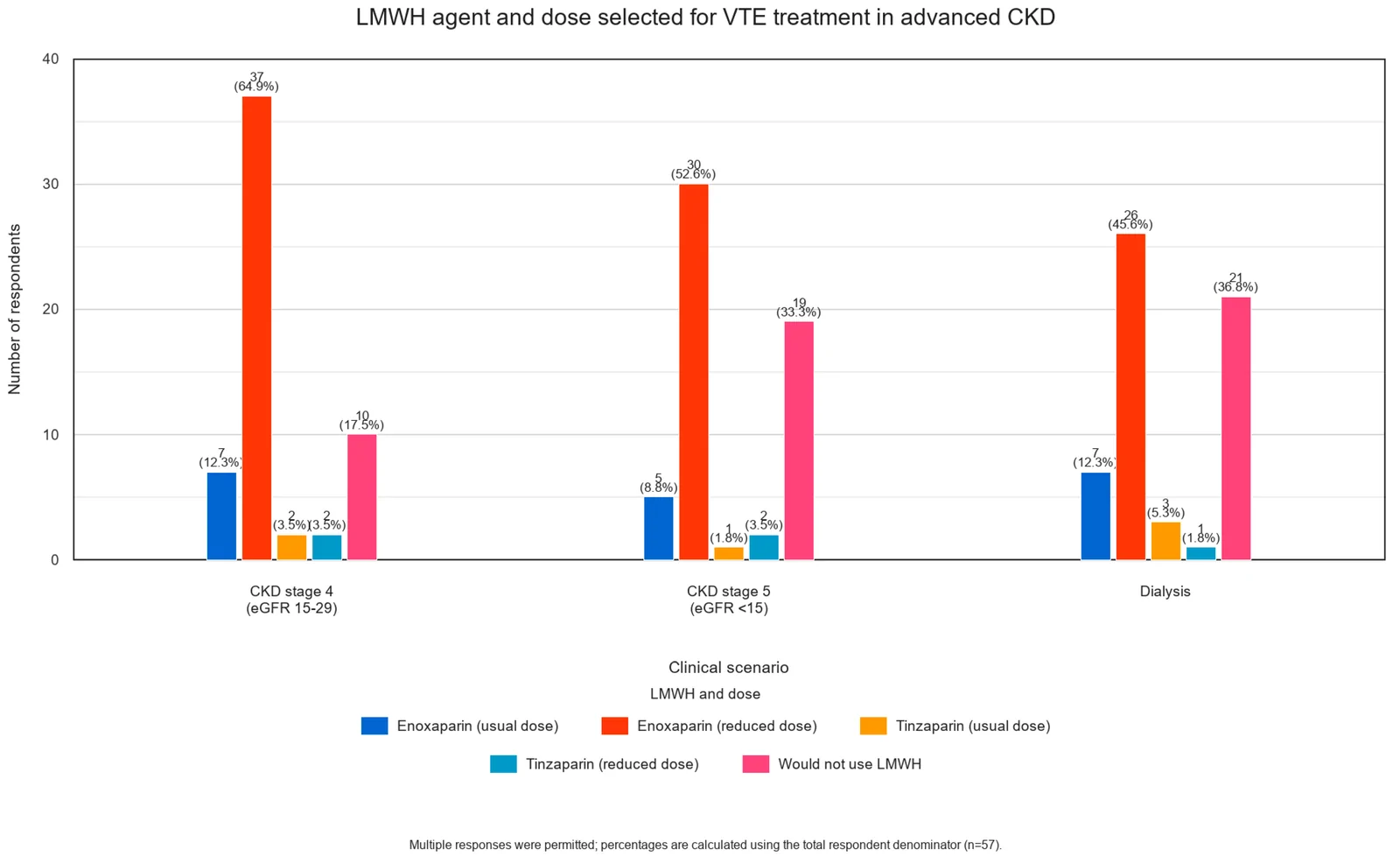
Type and dose of LMWH used for treatment of VTE in patients with CKD.

Type and dose of LMWH for VTE treatment: physicians most frequently selected reduced dose of enoxaparin, followed by avoiding use of LMWH (see Figure 7).

***Deviation across subgroup analysis:*** physicians with less than 10 years of experience selected more frequently not to use LMWH in patients with stage 5 CKD and patients on dialysis. Also, physicians in the nephrology track selected a reduced dose of enoxaparin in stage 4 CKD or in dialysis more frequently, while physicians in the hematology track opted not to use a reduced dose of enoxaparin.

**Figure 8:**
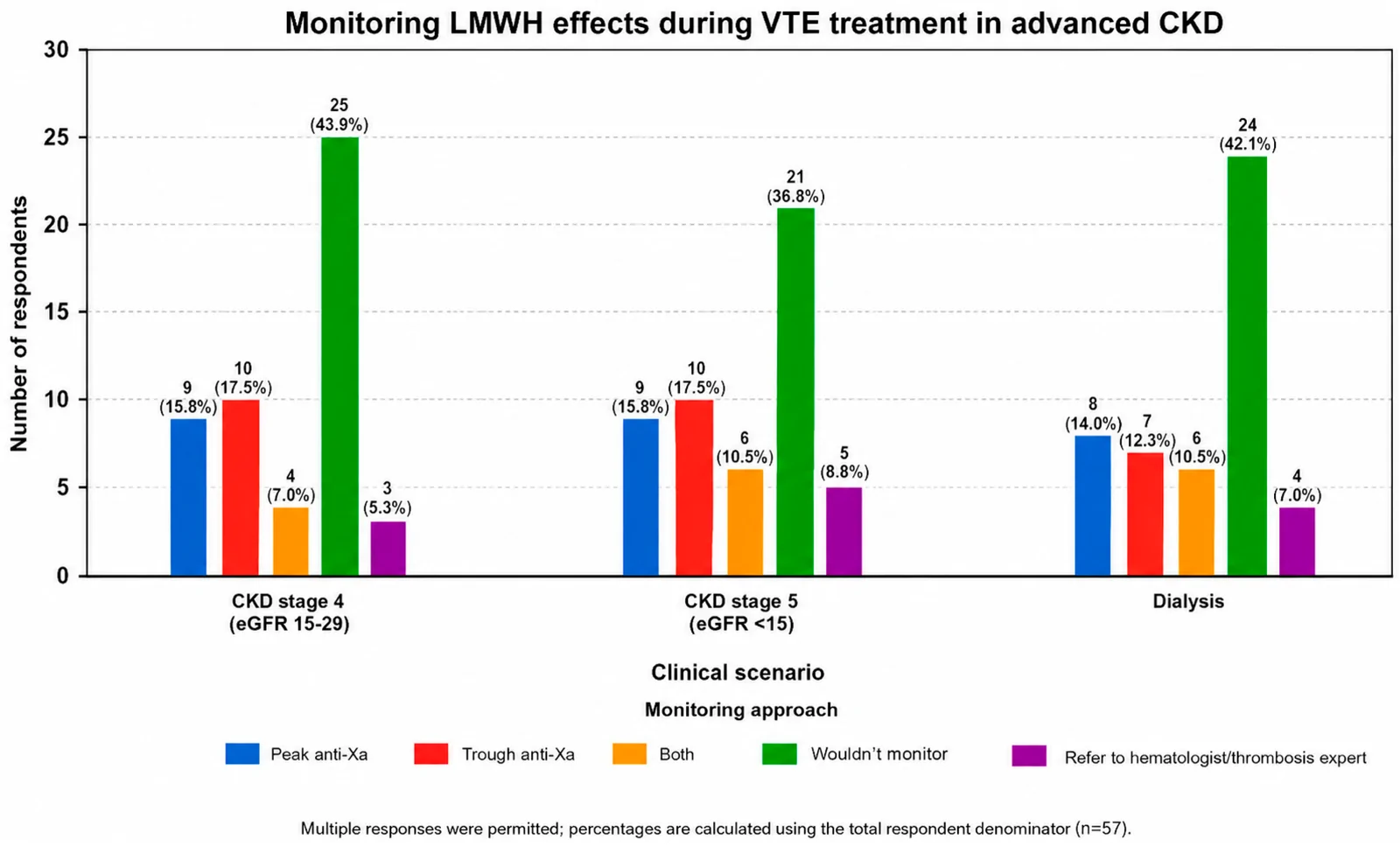
Type and dose of LMWH used for treatment of VTE in patients with CKD.

Monitoring LMWH effects during treatment: Physicians selected most frequently not to monitor LMWH anticoagulation effects by using anti-Xa levels (see Figure 8).

***Deviation across subgroup analysis:*** Variability in such practice was seen when responses were broken down by specialty, practice setting, and experience (see Table S6 in Supplementary Materials).

**Figure 9:**
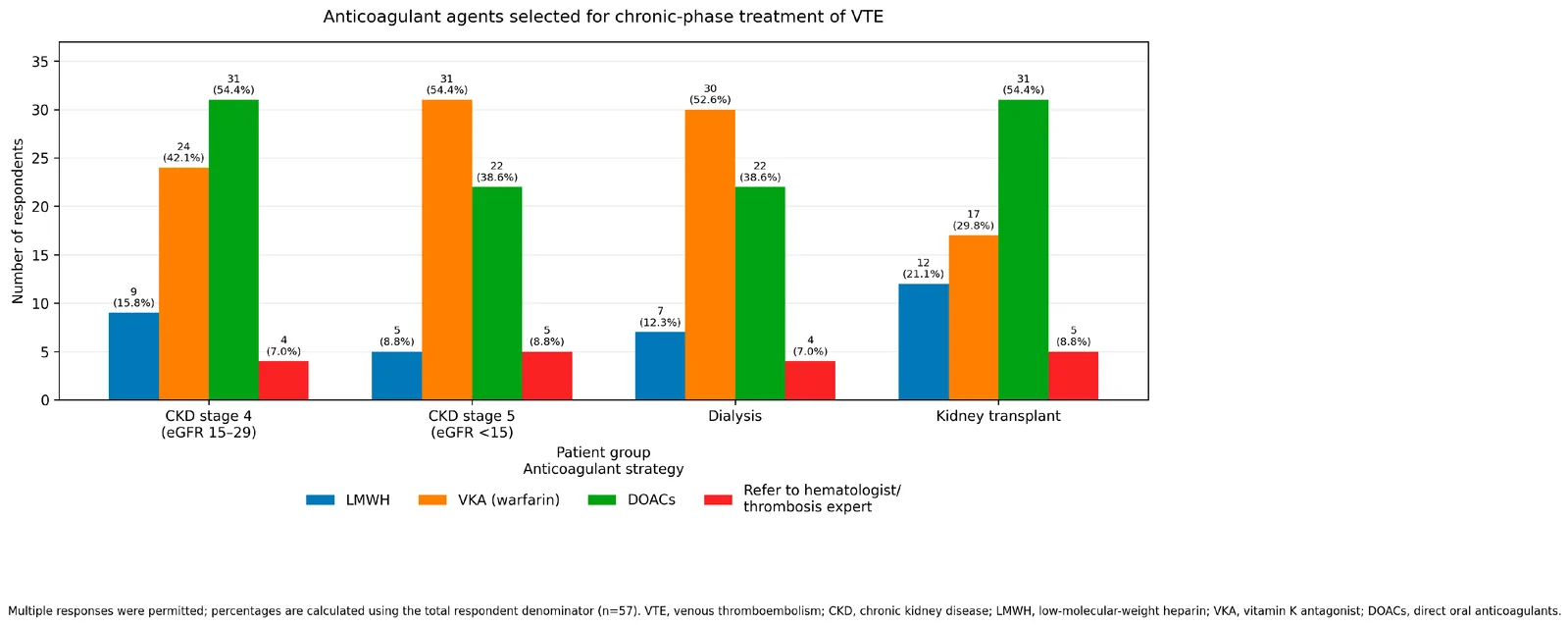
Anticoagulation agents used for chronic phase treatment of VTE (more than one option could be chosen).

Treatment of chronic VTE: warfarin was selected most frequently for patients with stage 5 CKD and dialysis, while DOAC was selected most frequently for patients with stage 4 CKD or kidney transplants. Referral to hematology/thrombosis experts was infrequently selected (see Figure 9).

***Deviation across subgroup analysis:*** Warfarin was selected more frequently by physicians with more than 10 years of experience for patients with kidney transplants, while physicians working in an outpatient dialysis setting selected warfarin more frequently for patients with stage 4 CKD and DOAC for patients on dialysis (see Table S7 in Supplementary Materials).

**Figure 10:**
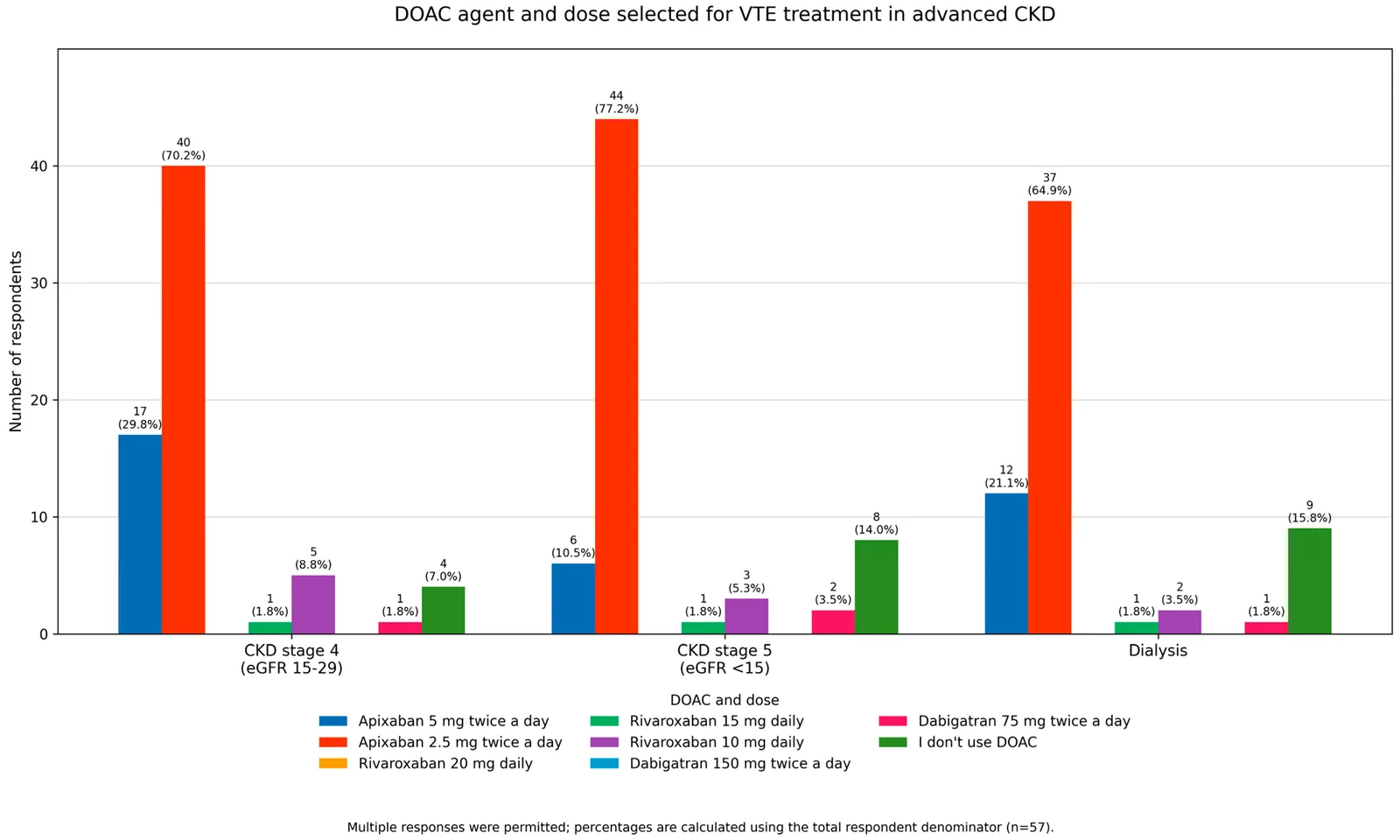
Type and dose of DOACs used during chronic phase treatment of VTE (more than one option could be chosen). (orange and light blue column: It indicates that no responses were recorded for such category.)

Dose of DOACs used during treatment of chronic VTE: apixaban 2.5 mg BID was selected most frequently in all stages of CKD, including dialysis (see Figure 10).

***Deviation across subgroup analysis:*** Apixaban 5 mg BID was selected more frequently by physicians working in dialysis units for patients with stage 4 CKD. Physicians in the hematology track selected Dabigatran 75 mg BID more frequently for patients on dialysis (see Table S8 in Supplementary Materials).

#### 3.3.4. Management of AF in Patients with CKD

**Figure 11:**
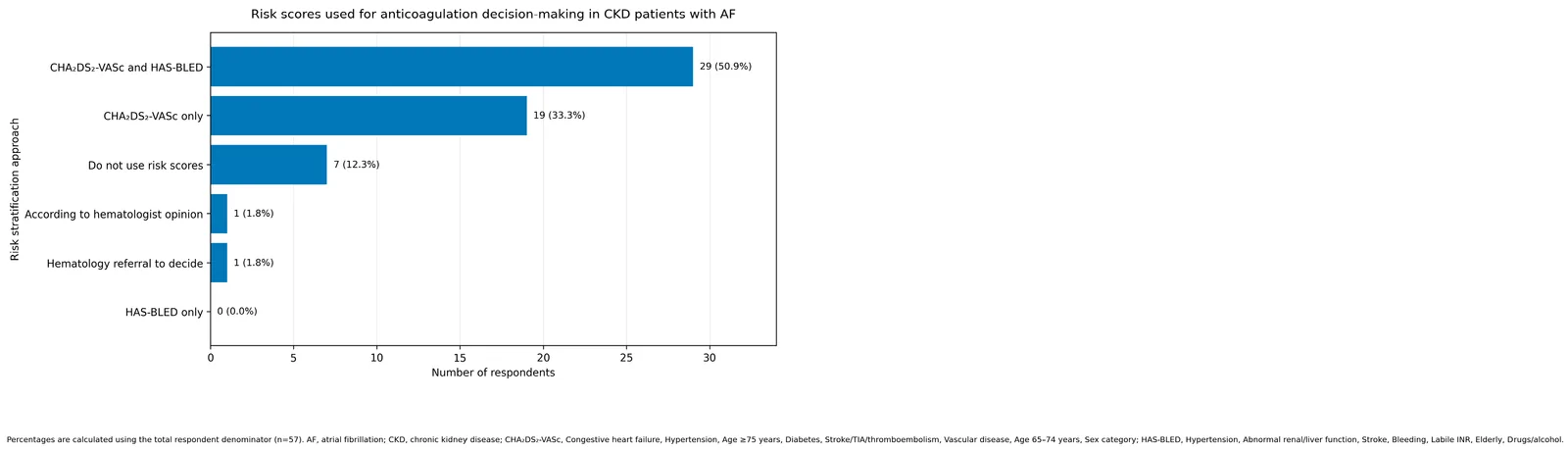
AF-related risk scores routinely used in decision-making to use anticoagulation in patients with CKD and AF.

Risk stratification: Half of respondents selected using both CHA_2_DS_2_-VASc and HAS-BLED scores when deciding anticoagulation use in patients with CKD and AF, while one third selected using CHA_2_DS_2_-VASc alone (see Figure 11).

***Deviation across subgroup analysis:*** physicians with experience of less than 10 years or in hematology track selected using both systems more frequently than their counterparts, and CHA_2_DS_2_-VASc alone was more frequently selected by physicians in outpatient dialysis units (see Table S9 in Supplementary Materials).

**Figure 12:**
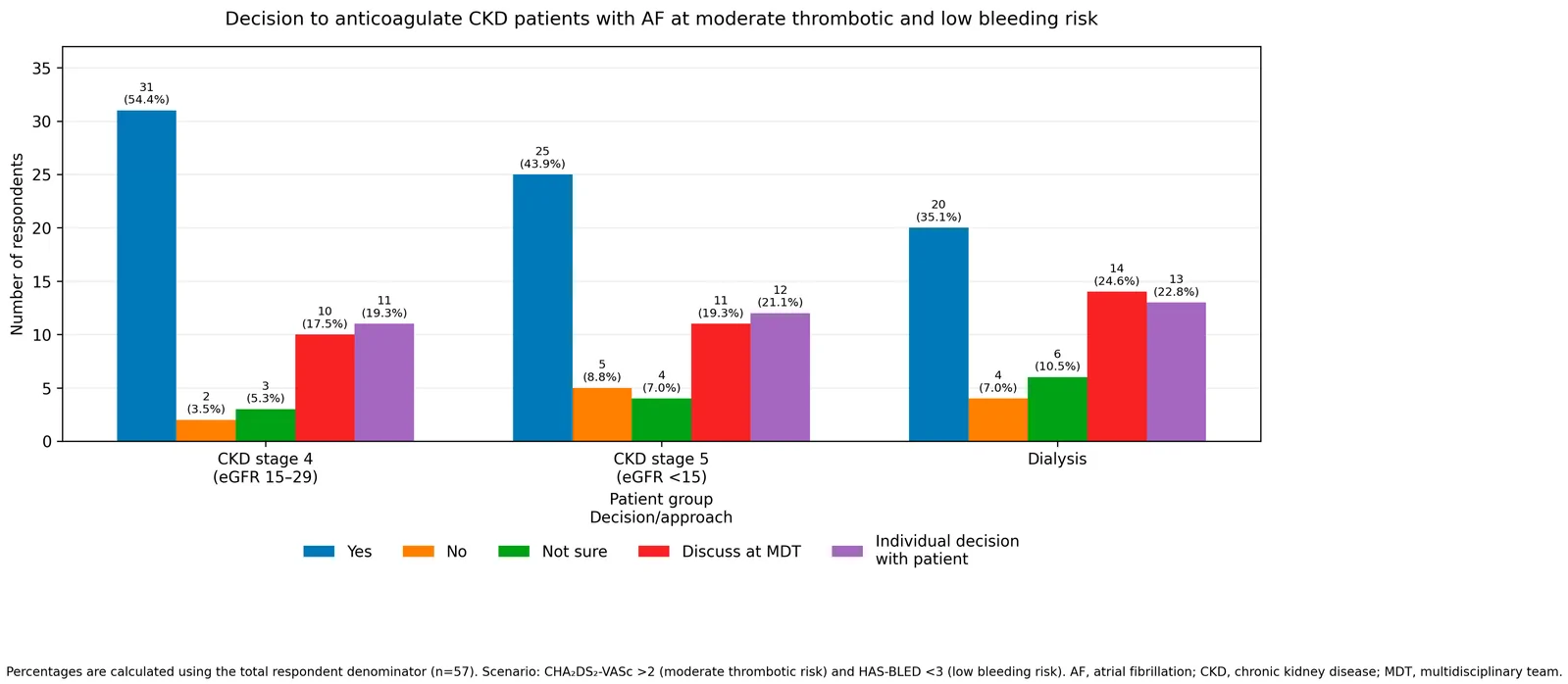
Decision to anticoagulate patients with AF and CKD who have CHADSVASC > 2 (moderate thrombosis risk) with HASBLED < 3 (low bleeding risk).

Decision about anticoagulant in patients with AF who are at *moderate-risk thrombosis and low-risk bleeding*: Physicians selected most frequently to anticoagulate across all strata of CKD (see Figure 12).

***Deviation across subgroup analysis:*** Physicians in the nephrology track, working in outpatient dialysis units, or those with more than 10 years of experience selected most frequently to discuss at MDT round for patients on dialysis (see Table S10 in Supplementary Materials).

**Figure 13:**
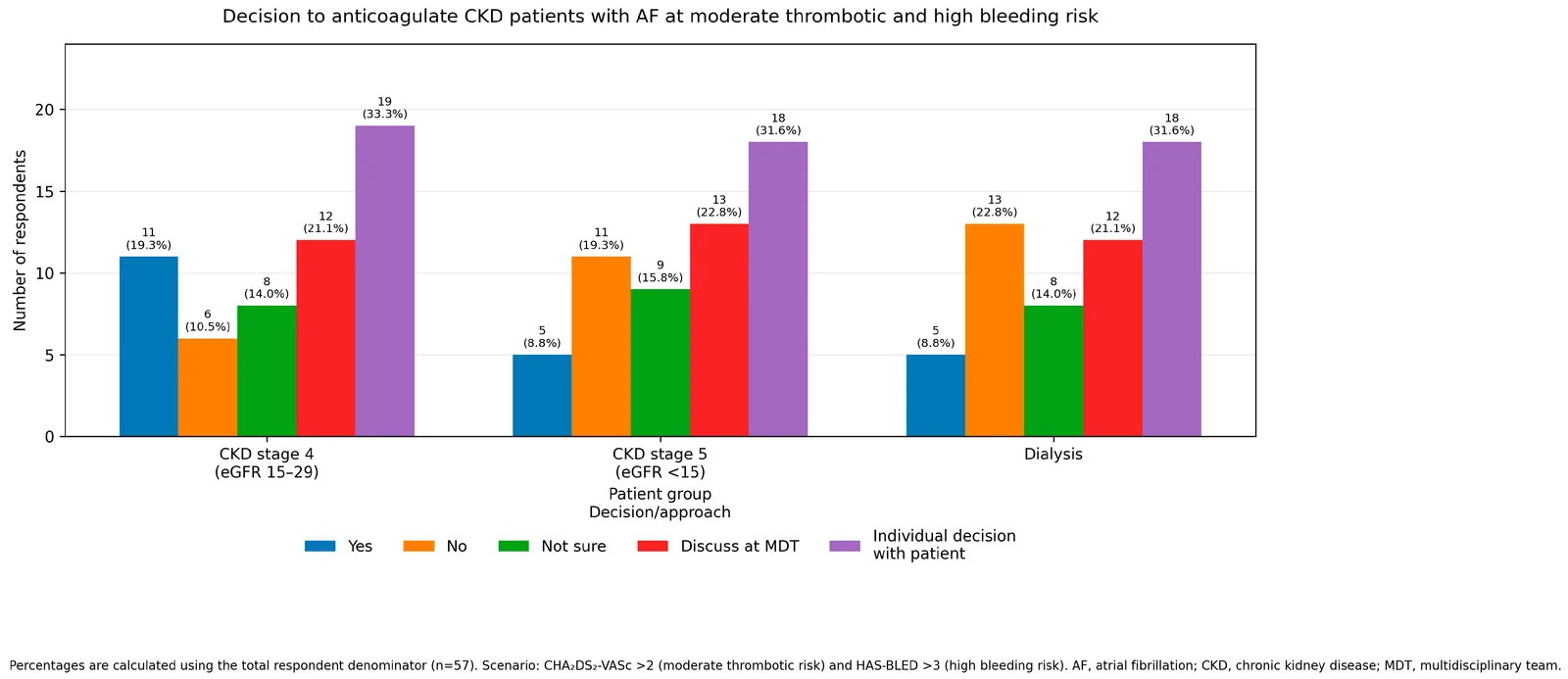
Decision to anticoagulate patients with AF and CKD who have CHADSVASC > 2 (moderate thrombosis risk) with HASBLED > 3 (high bleeding risk).

Decision about Anticoagulant in patients with AF who are at *moderate-risk thrombosis and high-risk bleeding*, physicians selected most frequently individualize decision making across all strata of CKD (see Figure 13).

***Deviation across subgroup analysis:*** Physicians with 5–10 years of experience opted not to anticoagulate if patients were on dialysis. Physicians in the hematology track selected uncertainty to anticoagulate most frequently in patients with Stage 5 CKD. Discussion at MDT meetings was most frequently selected by physicians working in private hospitals or in outpatient dialysis units across all strata of CKD (see Table S11 in Supplementary Materials).

**Figure 14:**
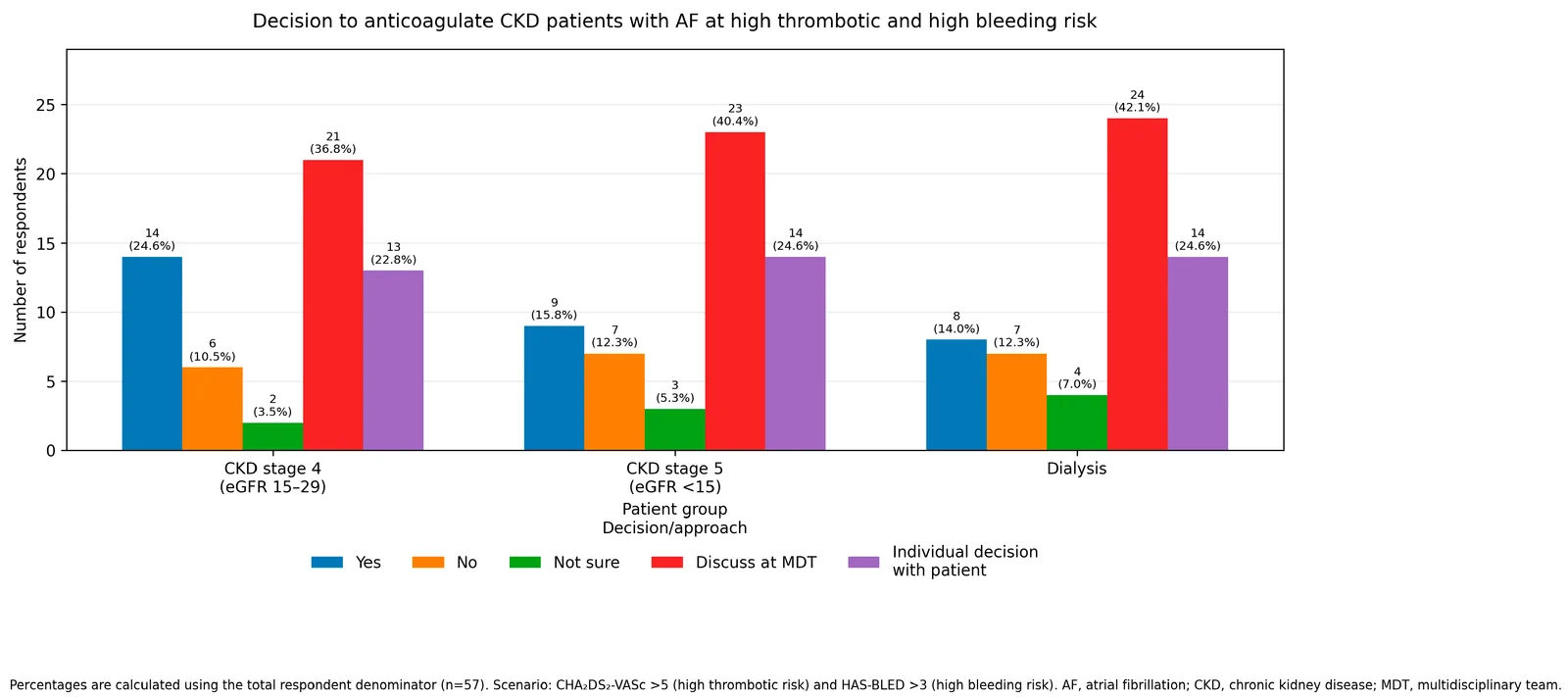
Decision to anticoagulate patients with AF and CKD who have a CHA2DS2-VASc score >5 (high thrombosis risk) and a HASBLED score >3 (high bleeding risk).

Decision about Anticoagulant in patients with AF in *high-risk thrombosis and high-risk bleeding settings*: respondents most frequently selected discussion at the MDT meeting across all CKD strata (see Figure 14).

***Deviation across subgroup analysis:*** Individualized decision making was most frequently selected by physicians for less than 5 years, and physicians in the hematology track selected most frequently to anticoagulate patients with Stage 4 CKD (see Table S12 in Supplementary Materials).

**Figure 15:**
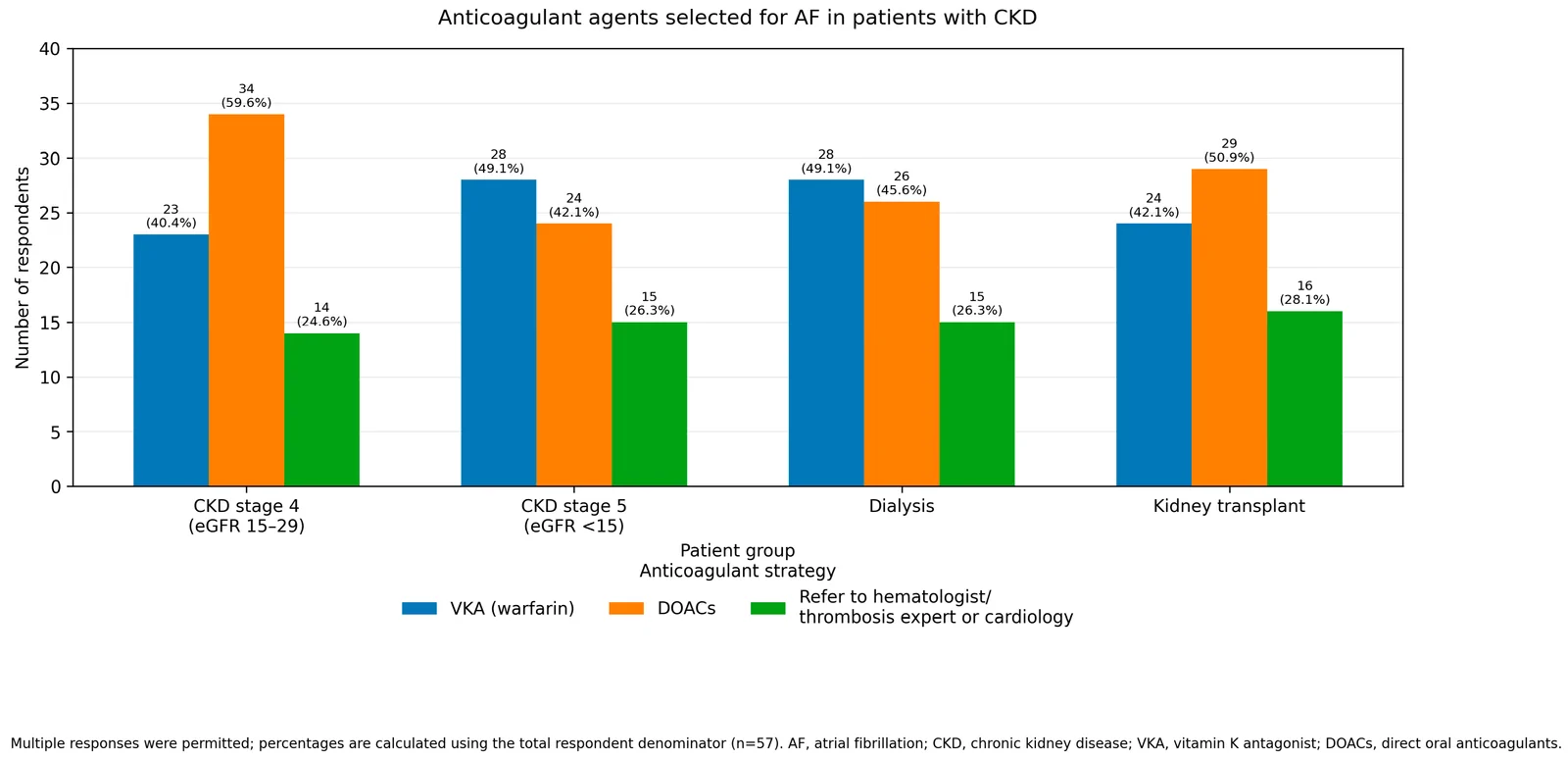
Anticoagulant agents used in patients with AF and CKD (more than one option could be chosen).

Type of anticoagulant used for management of AF: respondents chose DOACs more frequently, followed by warfarin for the treatment of AF in patients with Stage 4 CKD or those who underwent kidney transplant, and choosing warfarin more frequently followed by DOACs in patients with stage 5 CKD or on dialysis (see Figure 15).

***Deviation across subgroup analysis:*** Physicians working in private practice or physicians in the nephrology track choose DOACs over warfarin in patients with Stage 5 CKD disease or on dialysis. In addition, physicians in the nephrology track selected VKA most frequently in patients who underwent kidney transplantation (see Table S13 in Supplementary Materials).

**Figure 16:**
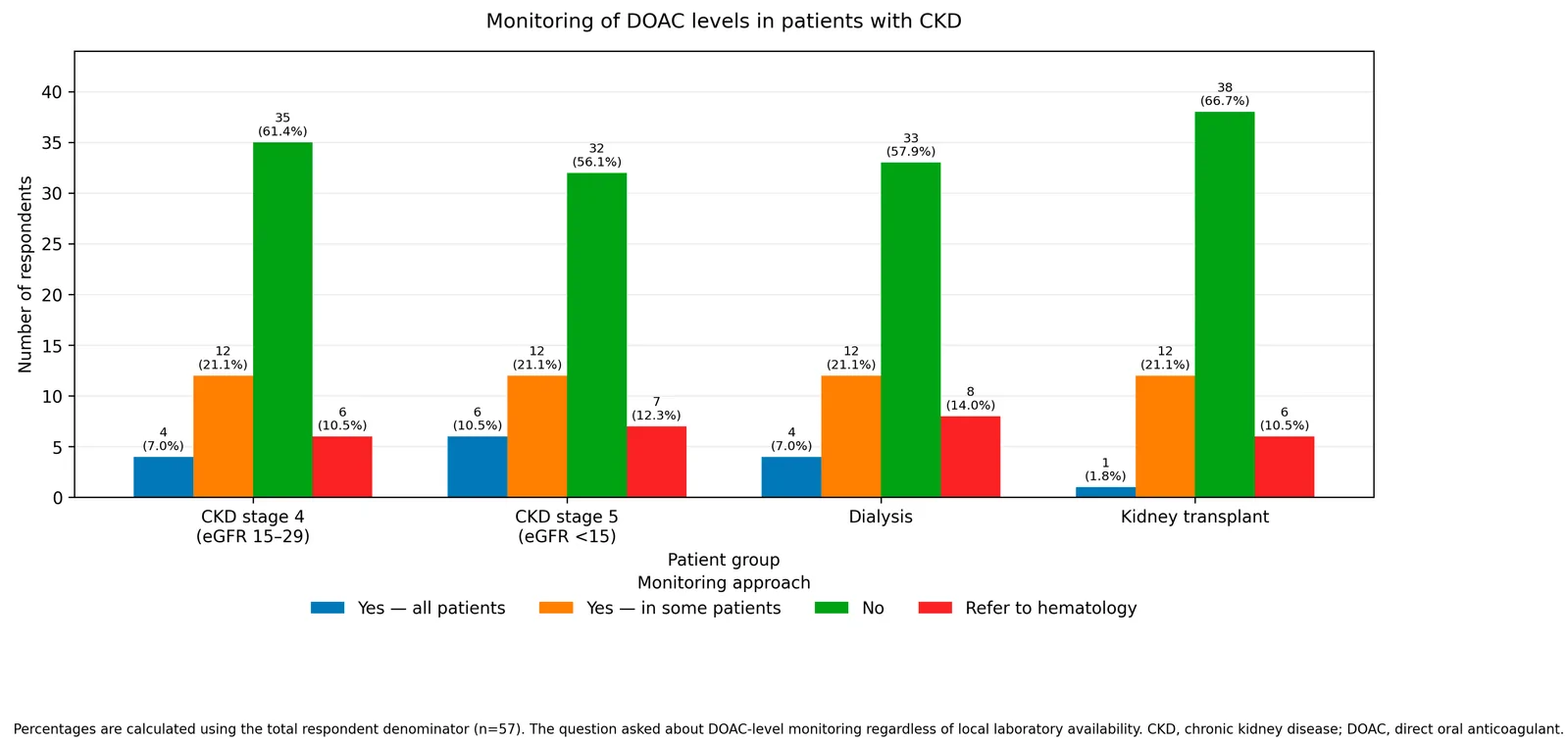
Monitoring DOAC levels in patients with CKD (regardless of the ability of the lab to perform such a test).

Dose and monitoring of DOACs in patients with CKD and AF: Apixaban (particularly 2.5 mg twice daily) was the most frequently reported DOAC regimen in patients with advanced CKD or on dialysis (see Table S14 in Supplementary Materials). The majority chose not to monitor DOAC levels in any advanced CKD settings, including dialysis, as well as in patients who underwent kidney transplant (see Figure 16 and Table S15 in Supplementary Materials).

#### 3.3.5. Anticoagulation in Nephrotic Syndrome

For nephrotic syndrome with normal kidney function, the most selected factors that influence decision regarding use of anticoagulation were: serum albumin, followed by bleeding risk, degree of proteinuria, and primary renal condition (see Table 2).

***Deviation across subgroup analysis:*** bleeding risk was a leading decision-related factor for physicians in the hematology track, and those with more than 10 years of experience (see Table S16 in Supplementary Materials).

In patients with nephrotic syndrome, normal renal function and serum albumin level <20 g/dL, respondents selected warfarin more frequently, followed by DOACs and LMWH (prophylactic or therapeutic dose). For the same scenario but with serum albumin 20–25 g/dL, warfarin or LMWH prophylactic dose was likely to be selected, followed by DOACs. For the same scenario but with serum albumin 25–30 g/dL, the majority chose not to use anticoagulation (see Table 3).

## 4. Discussion

Our survey highlights homogeneity as well as heterogeneity in aspects of anticoagulation practice in patients with advanced CKD. We noticed there was variation in measuring renal function according to specialty. Experts have recommended using the Chronic Kidney Disease Epidemiology (CKD-EPI) creatinine equation over other equations [9]. This is because other equations have limited precision and underestimate GFR when creatinine is highly elevated [10]. Underestimation or overestimation of GFR may affect the use of DOACs in patients with CKD, as their use is dependent on a certain threshold of CKD [11]. The variation in such practice may be due to DOAC trials, and many product labels have estimated creatinine clearance by Cockcroft–Gault, while nephrologists are more accustomed to using CKD-EPI for CKD staging. Stage 5 CKD.

The preference for using UFH for VTE prophylaxis in patients with stage 4 CKD, stage 5 CKD, or on dialysis likely reflects its short half-life, reversibility, clinician familiarity, and relative independence from renal clearance, making it an attractive option in patients at high bleeding risk or with rapidly changing renal function [12]. This pattern is highly consistent with recommendations [13]. By contrast, reduced-dose LMWH use decreased as kidney disease severity increased, consistent with concern about renal accumulation in advanced kidney disease [14]. The selection of DOACs for VTE prophylaxis by some physicians, particularly those working in outpatient dialysis settings, may be preferred due to their route of administration and lack of need for monitoring, but this is not fully consistent with current consensus, as patients with severe renal impairment were excluded from trials [15].

For treatment of acute VTE, UFH was the most frequently selected agent in patients with stage 5 CKD or those on dialysis, while in patients with stage 4 CKD, physicians selected UFH and DOACs equally. The use of DOACs in these patients may be encouraged by growing evidence, albeit largely based on non-randomized studies, that suggest use of DOACs may be safe in patients with moderate kidney disease [16]. It was also not surprising that apixaban was the dominant DOAC when clinicians opted to use a DOAC, as it can be used in patients with GFR > 15 mL/minute [17].

Decision factors for using anticoagulation in patients nephrotic syndrome were led by serum albumin, followed by bleeding risk, proteinuria, and primary renal condition. This is consistent with the KDIGO guideline, which emphasizes hypoalbuminemia (<20–25 g/L) as the strongest predictor of thrombotic risk [18]. The inclusion of DOACs reflects evolving practice despite the concern that the drug is protein-bound. Thus, its use is an area of controversy as there is limited evidence on the efficacy and safety of using DOACs in patients with nephrotic syndrome [19,20].

Presence of a MDT in anticoagulation management is essential because it reduces bleeding and thrombotic complications, improves adherence to guidelines, and enhances patient safety through coordinated stewardship. Errors can occur at prescribing, monitoring, or administration stages, making multidisciplinary oversight critical [21]. A Canadian cohort study found that a multidisciplinary thrombosis service reduced recurrent venous thromboembolism, major bleeding, and mortality compared with usual care [22]. The UK survey showed that only 3% of respondents had access to a dedicated MDT, and renal function assessment varied by specialty. Our survey found that two-thirds of respondents reported no structured MDT pathway and renal function estimation differed between nephrology and hematology respondents [8]. It is important to emphasize that the higher proportion of respondents reporting MDT access in our cohort compared with the UK survey is likely due to the differences in how MDT care was defined and the composition of our sample.

Our survey found that physicians used both CHA_2_DS_2_-VASc and HAS-BLED scores for risk stratification, consistent with guidelines regarding management of AF [23]. However, one-third of physicians used CHA_2_DS_2_-VASc alone, underestimating bleeding risk. For moderate thrombotic risk and low bleeding risk, anticoagulation was most frequently selected across CKD strata, guideline-aligned, though MDT referral was underutilized. In moderate thrombotic risk with high bleeding risk, individualized decision-making was most common, consistent with guideline emphasis on balancing risks, but subgroup variability showed conservative non-anticoagulation in dialysis and uncertainty among hematologists. In high thrombotic and high bleeding risk scenarios, MDT discussion was most frequently selected, though less experienced physicians leaned toward individualized decisions without MDT input. DOAC dosing in AF showed widespread use of apixaban 2.5 mg BID in advanced CKD/dialysis, which is inconsistent with guidelines, as this dose is only indicated for extended prophylaxis or when dose-reduction criteria are met. Most physicians did not monitor DOAC levels, supported by the absence of any strong recommendation to monitor these drugs in patients with advanced CKD [24].

As stated above, patients with creatinine clearance < 30 mL/min were not allowed to enroll in important randomized clinical trials that evaluated DOAC efficacy in patients with AF or VTE [16,25]. However, many non-randomized cohort studies and systematic reviews/meta-analysis have been published to address the uncertainty of using DOACs in patients receiving dialysis with advanced CKD [16,25]. For example, in the COMBINE AF patient-level meta-analysis, standard-dose DOACs were safer and more effective than warfarin down to a creatinine clearance of at least 25 mL/min, whereas lower-dose DOACs did not reduce bleeding and were associated with more stroke/systemic embolism and death [2]. Similarly, in a nationwide US cohort of non-dialysis Stage 4/5 CKD and AF, warfarin and rivaroxaban were both associated with more major bleeding than apixaban (hazard ratios 1.85 and 1.69, respectively), without a clear ischemic-stroke advantage over apixaban [4]. These data support the growing preference for apixaban in patients with AF and severe non-dialysis CKD. Thus, it was not surprising that DOACS (and in particular apixaban) were chosen more frequently as an anticoagulant in such a population. However, guidelines for the management of AF advise not to reduce the dose of DOACS unless patients meet specific criteria [26]. For apixaban, 2 out of 3 must be present for dose reduction: (1) age > 79, (2) body weight < 61 kg, (3) serum creatinine > 132 umol/L. It also recognized an evidence gap for using oral anticoagulants in patients with advanced CKD. Thus, the decision to choose apixaban 2.5 mg twice daily predominantly in our survey based on renal impairment exclusively likely reflects clinicians’ attitude as being cautious rather than following guidelines [2,4].

We found similarities and differences when comparing our results in this survey to other surveys published in other countries. For example, the UK nephrology/hematology survey found VKA was commonly preferred in advanced CKD and apixaban was the preferred DOAC when DOACs were used [8]. Similarly, in the Canadian survey, warfarin remained the preferred anticoagulant for 88.8% of respondents; only 37.9% would consider choosing a DOAC, and nephrologists were more likely to consider DOACs compared to cardiologists (55.8% vs. 32.4%) [27]; by contrast, in India, 93.8% preferred DOACs over warfarin and 94.9% preferred adjusted dosing [7].

This study has several strengths. (1) The survey captured real-world anticoagulation practices across a wide range of clinically relevant scenarios including VTE prophylaxis, acute and chronic VTE treatment, AF, and nephrotic syndrome. (2) Inclusion of clinicians from different specialties, levels of experience, and healthcare settings enabled meaningful subgroup analyses that identified drivers of practice variation. (3) The study explored several understudied practical aspects of care, including renal function estimation methods, DOAC dosing and monitoring strategies, and availability of multidisciplinary pathways. (4) The presence of hypothetical clinical scenarios enhanced the applicability of findings to routine clinical practice. (5) The regional nature of the study contributes valuable data. (6) The study identifies important targets for future prospective research and healthcare system improvement initiatives.

Nevertheless, this study also has several weaknesses. (1) It was a cross-sectional survey with a modest sample size, and therefore it is vulnerable to selection bias. (2) The exact number of clinicians who received the invitation could not be determined, and a response rate could not be calculated; because it was anonymous, identifying any differences between respondents and nonrespondents could not be attempted. (3) Respondents may have been clinicians with greater interest or experience in anticoagulation in CKD. In addition, nephrologists and government-hospital physicians were over-represented, which may have influenced the observed anticoagulant preferences and limits generalizability to all national practice settings. (4) Several subgroup comparisons by specialty, experience, and practice setting involved small numbers; in addition, the results themselves rely predominantly on nephrologists’ responses. (5) The hypothetical scenarios necessarily simplified real-world anticoagulation decisions and did not fully capture factors such as concomitant antiplatelet therapy, frailty, body weight, time in therapeutic range, local formulary restrictions, or access to drug-level monitoring. (6) The results were presented in a descriptive manner, and we did not attempt to do a statistical comparison as the goal of the survey was to explore the attitude pattern of physicians and our observations were hypothesis-generation rather than solid conclusions. (7) Responses may be affected by social desirability bias; clinicians may report what they perceive to be best practice rather than their actual prescribing behavior, particularly in controversial areas such as DOAC use and anticoagulation in dialysis. (8) Because the survey was conducted within a single national context, generalizability to other regions may be limited.

## 5. Conclusions

Anticoagulation practice in advanced CKD remains heterogeneous in some domains. Our findings support development of CKD-specific anticoagulation pathways and multidisciplinary decision-making models. Future research should prioritize pragmatic trials in advanced CKD as well as kidney transplant recipients and patients with nephrotic syndrome.

## Figures and Tables

**Table 1:** Baseline characteristics for the 57 participants.

Variable	n (Total n)	(%)
Years in practice		
Less than 5 years	17	29.8
5–10 years	15	26.3
More than 10 years	25	43.9
Education/Training		
Saudi Arabia	25	43.9
Canada	15	26.4
USA	6	10.5
UK	5	8.7
Master’s/PhD in Egypt	4	7
other regions/boards	12	21.1
Specialty		
Hematology Consultant/Specialist	18	31.6
Hematology In Training	2	3.5
Nephrology Consultant/Specialist	37	64.9
Practice Setting		
Government Hospital	34	59.6
Outpatient dialysis units	6	10.5
Private Hospital	6	10.5
Both	11	19.3
Type of exposure to CKD cases		
Patients with stage 4 & 5 CKD (eGFR < 30, NOT ON DIALYSIS) and acute venous thromboembolic event (VTE)	44	77.2
Patients receiving dialysis (Peritoneal or hemodialysis) with acute VTE	50	87.7
Patients with stage 4 & 5 CKD (eGFR < 30, NOT ON DIALYSIS) and atrial fibrillation (AF)	44	77.2
Patients receiving dialysis (PD or HD) patients with AF	45	78.9
Patients with stage 4 & 5 CKD (eGFR < 30, NOT ON DIALYSIS) and requiring medical VTE prophylaxis	41	71.9
Patients receiving dialysis (PD or HD) patients requiring medical VTE prophylaxis	46	80.7
Patients with kidney transplant	33	57.9
Patients with nephrotic syndrome	39	68.4
Presence of a structured multidisciplinary team to manage patients with CKD on anticoagulants		
Yes	14	24.6
No	38	66.7
Don’t know/not sure	5	8.8

**Table 2:** Factors that influence decisions about anticoagulation in nephrotic syndrome with normal kidney function (more than one option could be chosen).

Variable	Response (%)
Bleeding risk	39 (68.4)
Degree of proteinuria	31 (54.4)
I refer them to nephrologist, or hematologist (or thrombosis expert)	10 (17.4)
Primary renal condition	22 (38.6)
Serum albumin	42 (73.7)

**Table 3:** Type of anticoagulation agents in patients with nephrotic syndrome with normal kidney function and degree of hypoalbuminemia in g/dL (more than one option could be chosen).

Variable	Response (%)		
	Albumin < 20	Albumin 20–25	Albumin 26–30
DOAC	17 (29.8)	12 (21.1)	8 (14)
LMWH therapeutic dose	17 (29.8)	11 (19.3)	9 (15.8)
LMWH prophylactic dose	17 (29.8)	16 (28.1)	8 (14)
Aspirin	7 (12.3)	7 (12.3)	5 (8.8)
Warfarin	22 (38.6)	16 (28.1)	10 (17.5)
None	1 (1.8)	10 (17.5)	27 (47.4)
I refer them to nephrologist	8 (14)	7 (12.3)	6 (10.5)
I refer them to hematologist/thrombosis expert	7 (12.3)	4 (7.1)	3 (5.3 %)

## Data Availability

The data that support the findings of this study are not openly available due to reasons of sensitivity and are available from the corresponding author upon reasonable request.

## Supplementary Materials

The following supporting information can be downloaded at: https://www.mdpi.com/article/10.3390/jcm15176517/s1. Table S1: Assessment of renal function when using anticoagulants in CKD; Table S2: Type pharmacological VTE PROPHYLAXIS used in patients with CKD at various stages (more than one option could be chosen); Table S3: Anticoagulation agents alternative to heparin for VTE PROPHYLAXIS in patients with CKD at various stages; Table S4: Anticoagulation agents used for acute phase treatment of VTE; Table S5: type and dose of LMWH for VTE treatment; Table S6: Monitoring of LMWH during VTE treatment; Table S7: Anticoagulation agents used for chronic phase treatment of VTE; Table S8: DOACs for treatment of VTE which DOAC and what dose would you use; Table S9: Atrial fibrillation related risk scores routinely used in decision making when anticoagulating CKD patients with AF; Table S10: Decision to anticoagulate patients with Afib and CKD who have CHADSVASC > 2 (moderate thrombosis risk) with HASBLED < 3 (low bleeding risk). Table S11: Decision to anticoagulate patients with Afib and CKD who have CHADSVASC>2 (moderate thrombosis risk) with HASBLED>3 (high bleeding risk). Table S12: Decision to anticoagulate patients with Afib and CKD who have anticoagulate CHADSVASC>5 (high thrombosis risk) with HASBLED>3 (high bleeding risk). Table S13: anticoagulants agents used in patients with AF and CKD (more than one option could be chosen). Table S14: type and dose of DOACs used in patients with AF and CKD (more than one option could be chosen). Table S15: monitoring DOAC levels in patietns with CKD (regardless of the ability of lab to perform such a test). Table S16: factors that influence decisions about anticoagulation in nephrotic syndrome with normal kidney function (more than one option could be chosen). File S1. Survey Questionnaire and Response Options.
