# Supplementary material for: A Survey on Anticoagulation Practices in Patients with Chronic Kidney Disease in Saudi Arabia
Source: J Clin Med. 2026 Aug 23;15(17):6517. doi: 10.3390/jcm15176517 (PMC13566149; doi:10.3390/jcm15176517)
Supplement: Supplementary file 1 [file jcm-15-06517-s001.zip › jcm-4340944-File S1.pdf]

Supplementary File S1. Survey Questionnaire and Response Options

Anticoagulation in Patients with Chronic Kidney Disease (CKD)

Part 1. Respondent characteristics and practice setting

1. What is your job role? (According to the Saudi Council classification)

Response format: Single choice.

Answer options:
- Nephrology Consultant
- Hematology Consultant
- Nephrology Specialist (or equivalent, e.g., registrar)
- Hematology Specialist (or equivalent, e.g., registrar)
- Nephrology Fellow in training
- Hematology Fellow in training

2. Please indicate where your fellowship training was in nephrology or hematology.

Response format: Multiple choice.

Answer options:
- Saudi Board in Hematology or Nephrology
- Arab Board
- Jordanian Board
- American Board of Internal Medicine (or Fellowship in USA)
- Royal College of Physicians and Surgeons of Canada (or Fellowship in Canada)
- Royal Colleges of Physicians of the UK (MRCP) (or Fellowship in UK)
- Masters or PhD / Fellowship in Egypt
- Fellowship training in India or Pakistan
- Other

3. Please indicate your gender.

Response format: Single choice.

Answer options:
- Male
- Female

4. Please indicate the number of years you have been practising nephrology or hematology.

Response format: Single choice.

Answer options:
- Less than 5 years
- 5-10 years
- More than 10 years

5. Please indicate your primary practice setting.

Response format: Single choice.

Answer options:
- Government Hospital
- Private Hospital
- Both
- Outpatient dialysis units only

6. As part of your job role, would you see any of the below patients?

Response format: Multiple choice.

Answer options:

- CKD stage 4 and 5 (eGFR <30, not on dialysis) patients with acute venous thromboembolic event (VTE)
- Dialysis patients (peritoneal dialysis [PD] or hemodialysis [HD]) with acute VTE
- CKD stage 4 and 5 (eGFR <30, not on dialysis) patients with atrial fibrillation (AF)
- Dialysis patients (PD or HD) with AF
- CKD stage 4 and 5 (eGFR <30, not on dialysis) patients requiring medical VTE prophylaxis
- Dialysis patients (PD or HD) requiring medical VTE prophylaxis
- Patients with kidney transplant
- Patients with nephrotic syndrome

7. Does your centre have a structured multidisciplinary team to manage patients with CKD on anticoagulants?

Response format: Single choice.

Answer options:
- Yes
- No
- I don't know/not sure

Part 2. VTE prophylaxis in CKD

8. How would you assess renal function in the context of prescribing the following anticoagulants in CKD?

Response format: Matrix; select one most-used method per anticoagulant class.

Rows:
- DOACs
- LMWH
- Fondaparinux
- Unfractionated heparin

Column/answer options:
- Cockcroft-Gault
- Modification of Diet in Renal Disease (MDRD) equation
- Chronic Kidney Disease Epidemiology Collaboration (CKD-EPI) equation

9. What do you routinely use for pharmacological VTE prophylaxis in CKD stage 4 (eGFR 15-29) patients?

Response format: Multiple choice.

Answer options:
- Enoxaparin 40 mg subcutaneously once daily
- Enoxaparin 20-30 mg subcutaneously once daily
- Dalteparin 5,000 units subcutaneously once daily
- Tinzaparin 4,500 units subcutaneously once daily
- Unfractionated heparin 5,000 units subcutaneously every 8 to 12 hours

10. What do you routinely use for pharmacological VTE prophylaxis in CKD stage 5 (eGFR <15) patients?

Response format: Multiple choice.

Answer options:
- Enoxaparin 40 mg subcutaneously once daily
- Enoxaparin 20-30 mg subcutaneously once daily
- Dalteparin 5,000 units subcutaneously once daily
- Tinzaparin 4,500 units subcutaneously once daily
- Unfractionated heparin 5,000 units subcutaneously every 8 to 12 hours

11. What do you routinely use for pharmacological VTE prophylaxis in dialysis patients?

Response format: Multiple choice.

Answer options:
- Enoxaparin 40 mg subcutaneously once daily
- Enoxaparin 20-30 mg subcutaneously once daily
- Dalteparin 5,000 units subcutaneously once daily
- Tinzaparin 4,500 units subcutaneously once daily

- Unfractionated heparin 5,000 units subcutaneously every 8 to 12 hours

12. If a patient with CKD was unable to have heparins, what option would you consider for VTE prophylaxis?

Response format: Matrix; multiple choices permitted for each patient group.

Rows:

- CKD stage 4 (eGFR 15-29)
- CKD stage 5 (eGFR <15)
- Dialysis

Column/answer options:

- Fondaparinux
- DOACs
- Mechanical prophylaxis
- Others
- None

Part 3. Acute and chronic VTE treatment in CKD

13. For patients with CKD, which options would you use for acute-phase treatment of VTE?

Response format: Matrix; multiple choices permitted for each patient group.

Rows:

- CKD stage 4 (eGFR 15-29)
- CKD stage 5 (eGFR <15)
- Dialysis
- Kidney transplant

Column/answer options:

- LMWH
- Unfractionated heparin
- DOACs
- Refer to Hematologist or thrombosis expert

14. If you use LMWH for VTE treatment, which LMWH would you use and at what dose?

Response format: Matrix; multiple choices permitted for each patient group.

Rows:

- CKD stage 4 (eGFR 15-29)
- CKD stage 5 (eGFR <15)
- Dialysis

Column/answer options:

- Enoxaparin (usual dose)
- Enoxaparin (reduced dose)
- Tinzaparin (usual dose)
- Tinzaparin (reduced dose)
- Dalteparin (usual dose)
- Dalteparin (reduced dose)
- I do not use LMWH

15. If you use LMWH for VTE treatment, would you monitor?

Response format: Matrix; select one option per patient group.

Rows:

- CKD stage 4 (eGFR 15-29)
- CKD stage 5 (eGFR <15)
- Dialysis

Column/answer options:

- Peak anti-Xa
- Trough anti-Xa
- Both

- Would not monitor
- Refer to Hematologist or thrombosis expert

16. For patients with CKD, which options would you use for chronic treatment of VTE?

Response format: Matrix; multiple choices permitted for each patient group.

Rows:

- CKD stage 4 (eGFR 15-29)
- CKD stage 5 (eGFR <15)
- Dialysis
- Kidney transplant

Column/answer options:

- LMWH
- VKA (warfarin)
- DOAC
- Refer to Hematologist or thrombosis expert

17. If you use DOACs for treatment of VTE, which DOAC and what dose would you use?

Response format: Matrix; multiple choices permitted for each patient group.

Rows:

- CKD stage 4 (eGFR 15-29)
- CKD stage 5 (eGFR <15)
- Dialysis

Column/answer options:

- Apixaban 5 mg twice daily
- Apixaban 2.5 mg twice daily
- Rivaroxaban 20 mg daily
- Rivaroxaban 15 mg daily
- Rivaroxaban 10 mg daily
- Dabigatran 150 mg twice daily

Part 4. Atrial fibrillation in CKD

18. What risk scores do you routinely use to aid your decision-making when anticoagulating CKD patients with AF?

Response format: Single choice.

Answer options:

- HAS-BLED
- CHA2DS2-VASc
- Both
- Don't use risk scores
- Other

19. Would you anticoagulate a patient with CHA2DS2-VASc >2 (moderate thrombosis risk) and HAS-BLED <3 (low bleeding risk)?

Response format: Matrix; select one option per patient group.

Rows:

- CKD stage 4 (eGFR 15-29)
- CKD stage 5 (eGFR <15)
- Dialysis

Column/answer options:

- Yes
- No
- Not sure
- Discuss at multidisciplinary team (MDT) round
- Individual decision made with patient

20. Would you anticoagulate a patient with CHA2DS2-VASc >2 (moderate thrombosis risk) and HAS-BLED >3 (high bleeding risk)?

Response format: Matrix; select one option per patient group.

Rows:

- CKD stage 4 (eGFR 15-29)
- CKD stage 5 (eGFR <15)
- Dialysis

Column/answer options:

- Yes
- No
- Not sure
- Discuss at MDT
- Individual decision made with patient

21. Would you anticoagulate a patient with CHA2DS2-VASc >5 (high thrombosis risk) and HAS-BLED >3 (high bleeding risk)?

Response format: Matrix; select one option per patient group.

Rows:

- CKD stage 4 (eGFR 15-29)
- CKD stage 5 (eGFR <15)
- Dialysis

Column/answer options:

- Yes
- No
- Not sure
- Discuss at MDT
- Individual decision made with patient

22. What anticoagulants would you use for CKD patients with AF?

Response format: Matrix; multiple choices permitted for each patient group.

Rows:

- CKD stage 4 (eGFR 15-29)
- CKD stage 5 (eGFR <15)
- Dialysis
- Kidney transplant

Column/answer options:

- VKA (warfarin)
- DOACs
- Refer to Hematologist (or thrombosis expert) or Cardiology

23. If you use DOACs in patients with CKD and AF, what DOAC and dose do you use?

Response format: Matrix; multiple choices permitted for each patient group.

Rows:

- CKD stage 4 (eGFR 15-29)
- CKD stage 5 (eGFR <15)
- Dialysis

Column/answer options:

- Apixaban 5 mg twice daily
- Apixaban 2.5 mg twice daily
- Rivaroxaban 20 mg daily
- Rivaroxaban 15 mg daily
- Rivaroxaban 10 mg daily
- Dabigatran 150 mg twice daily

24. Would you check or monitor DOAC levels, regardless of the ability of the laboratory to perform such a test?

Response format: Matrix; select one option per patient group.

Rows:
- CKD stage 4 (eGFR 15-29)
- CKD stage 5 (eGFR <15)
- Dialysis
- Kidney transplant

Column/answer options:
- Yes - all patients
- Yes - in some patients
- No
- Refer to Hematology

Part 5. Nephrotic syndrome

25. What factors do you consider when making decisions about anticoagulation in nephrotic syndrome with normal kidney function?

Response format: Multiple choice.

Answer options:
- Degree of proteinuria
- Serum albumin
- Primary renal condition
- Bleeding risk
- Refer to Nephrologist or Hematologist (or thrombosis expert)

26. What options would you use for anticoagulation in a patient with nephrotic syndrome and serum albumin <20 g/dL with normal kidney function?

Response format: Multiple choice.

Answer options:
- DOAC
- LMWH therapeutic dose
- LMWH prophylactic dose
- Aspirin
- Warfarin
- None
- Refer to Nephrologist
- Refer to Hematologist (or thrombosis expert)

27. What options would you use for anticoagulation in a patient with nephrotic syndrome and serum albumin 20-25 g/dL with normal kidney function?

Response format: Multiple choice.

Answer options:
- DOAC
- LMWH therapeutic dose
- LMWH prophylactic dose
- Aspirin
- Warfarin
- None
- Refer to Nephrologist
- Refer to Hematologist (or thrombosis expert)

28. What options would you use for anticoagulation in a patient with nephrotic syndrome and serum albumin 26-30 g/dL with normal kidney function?

Response format: Multiple choice.

Answer options:

- DOAC
- LMWH therapeutic dose
- LMWH prophylactic dose
- Aspirin
- Warfarin
- None
- Refer to Nephrologist
- Refer to Hematologist (or thrombosis expert)
