# Supplementary material for: A Survey on Anticoagulation Practices in Patients with Chronic Kidney Disease in Saudi Arabia
Source: J Clin Med. 2026 Aug 23;15(17):6517. doi: 10.3390/jcm15176517 (PMC13566149; doi:10.3390/jcm15176517)
Supplement: Supplementary file 1 [file jcm-15-06517-s001.zip › jcm-4340944-supplementary.pdf]

Variable	YEARS EXPERIANCE			PRACTICE SETTING				SPECIALITY	
	5-10 years	< 5 years	> 10 years	Both	Government Hospital	Outpatient dialysis units	Private Hospital	Hematology track	Nephrology track
**DOACS**									
Cockcroft-gault	7 (46.7%)	10 (58.8%)	12 (48.0%)	6 (54.5%)	19 (55.9%)	3 (50.0%)	1 (16.7%)	18 (90.0%)	11 (29.7%)
The Chronic Kidney Disease Epidemiology Collaboration (CKD-EPI) equation	6 (40.0%)	4 (23.5%)	6 (24.0%)	2 (18.2%)	10 (29.4%)	2 (33.3%)	2 (33.3%)	0 (0.0%)	16 (43.2%)
The Modification of Diet in Renal Disease (MDRD) equation	6 (40.0%)	3 (17.6%)	8 (32.0%)	3 (27.3%)	9 (26.5%)	2 (33.3%)	3 (50.0%)	2 (10.0%)	15 (40.5%)
**LMWH**									
Cockcroft-gault	7 (46.7%)	9 (52.9%)	14 (56.0%)	7 (63.6%)	19 (55.9%)	3 (50.0%)	1 (16.7%)	19 (95.0%)	11 (29.7%)
The Chronic Kidney Disease Epidemiology Collaboration (CKD-EPI) equation	6 (40.0%)	4 (23.5%)	5 (20.0%)	1 (9.1%)	10 (29.4%)	2 (33.3%)	2 (33.3%)	0 (0.0%)	15 (40.5%)
The Modification of Diet in Renal Disease (MDRD) equation	6 (40.0%)	4 (23.5%)	7 (28.0%)	3 (27.3%)	9 (26.5%)	2 (33.3%)	3 (50.0%)	1 (5.0%)	16 (43.2%)
**FONDAPARINUX**									
Cockcroft-gault	7 (46.7%)	9 (52.9%)	11 (44.0%)	5 (45.5%)	18 (52.9%)	3 (50.0%)	1 (16.7%)	18 (90.0%)	9 (24.3%)
The Chronic Kidney Disease Epidemiology Collaboration (CKD-EPI) equation	6 (40.0%)	4 (23.5%)	5 (20.0%)	1 (9.1%)	10 (29.4%)	2 (33.3%)	2 (33.3%)	0 (0.0%)	15 (40.5%)
The Modification of Diet in Renal Disease (MDRD) equation	6 (40.0%)	4 (23.5%)	10 (40.0%)	5 (45.5%)	10 (29.4%)	2 (33.3%)	3 (50.0%)	2 (10.0%)	18 (48.6%)
**UFH**									
Cockcroft-gault	7 (46.7%)	9 (52.9%)	13 (52.0%)	6 (54.5%)	19 (55.9%)	3 (50.0%)	1 (16.7%)	19 (95.0%)	10 (27.0%)
The Chronic Kidney Disease Epidemiology Collaboration (CKD-EPI) equation	6 (40.0%)	6 (35.3%)	5 (20.0%)	1 (9.1%)	12 (35.3%)	2 (33.3%)	2 (33.3%)	1 (5.0%)	16 (43.2%)
The Modification of Diet in Renal Disease (MDRD) equation	6 (40.0%)	4 (23.5%)	8 (32.0%)	4 (36.4%)	9 (26.5%)	2 (33.3%)	3 (50.0%)	2 (10.0%)	16 (43.2%)

Table S1: Assessment of renal function when using anticoagulants in CKD

Variable	YEARS EXPERIANCE			PRACTICE SETTING				SPECIALITY	
	**5-10 years**	**< 5 years**	**> 10 years**	**Both**	**Government Hospital**	**Outpatient dialysis units ONLY**	**Private Hospital**	**Hematology track**	**Nephrology track**
**[CKD stage 4 (eGFR 15-29)]**									
Dalteparin 5,000 units SubQ once daily	4 (26.7%)	2 (11.8%)	0 (0.0%)	2 (18.2%)	3 (8.8%)	1 (16.7%)	0 (0.0%)	2 (10.0%)	4 (10.8%)
Enoxaparin 20-30 mg SubQ once daily	8 (53.3%)	6 (35.3%)	6 (24.0%)	1 (9.1%)	15 (44.1%)	2 (33.3%)	2 (33.3%)	7 (35.0%)	13 (35.1%)
Enoxaparin 40 mg SubQ once daily	3 (20.0%)	1 (5.9%)	9 (36.0%)	4 (36.4%)	6 (17.6%)	2 (33.3%)	1 (16.7%)	4 (20.0%)	9 (24.3%)
Tinzaparin 4,500 units SubQ once daily	1 (6.7%)	0 (0.0%)	1 (4.0%)	1 (9.1%)	1 (2.9%)	0 (0.0%)	0 (0.0%)	2 (10.0%)	0 (0.0%)
Unfractionated heparin 5,000 units SubQ every 8 to 12 hours	12 (80.0%)	13 (76.5%)	16 (64.0%)	6 (54.5%)	28 (82.4%)	2 (33.3%)	5 (83.3%)	15 (75.0%)	26 (70.3%)
**[CKD stage 5 (eGFR <15)]**									
Dalteparin 5,000 units SubQ once daily	3 (20.0%)	1 (5.9%)	0 (0.0%)	1 (9.1%)	2 (5.9%)	1 (16.7%)	0 (0.0%)	1 (5.0%)	3 (8.1%)
Enoxaparin 20-30 mg SubQ once daily	5 (33.3%)	4 (23.5%)	6 (24.0%)	2 (18.2%)	10 (29.4%)	1 (16.7%)	2 (33.3%)	3 (15.0%)	12 (32.4%)
Enoxaparin 40 mg SubQ once daily	3 (20.0%)	0 (0.0%)	5 (20.0%)	3 (27.3%)	3 (8.8%)	2 (33.3%)	0 (0.0%)	3 (15.0%)	5 (13.5%)
Tinzaparin 4,500 units SubQ once daily	1 (6.7%)	0 (0.0%)	1 (4.0%)	1 (9.1%)	1 (2.9%)	0 (0.0%)	0 (0.0%)	2 (10.0%)	0 (0.0%)
Unfractionated heparin 5,000 units SubQ every 8 to 12 hours	12 (80.0%)	15 (88.2%)	16 (64.0%)	7 (63.6%)	29 (85.3%)	2 (33.3%)	5 (83.3%)	16 (80.0%)	27 (73.0%)
**[Dialysis]**									
Dalteparin 5,000 units SubQ once daily	3 (20.0%)	0 (0.0%)	0 (0.0%)	1 (9.1%)	1 (2.9%)	1 (16.7%)	0 (0.0%)	1 (5.0%)	2 (5.4%)
Enoxaparin 20-30 mg SubQ once daily	6 (40.0%)	2 (11.8%)	3 (12.0%)	1 (9.1%)	8 (23.5%)	0 (0.0%)	2 (33.3%)	2 (10.0%)	9 (24.3%)
Enoxaparin 40 mg SubQ once daily	3 (20.0%)	0 (0.0%)	7 (28.0%)	2 (18.2%)	6 (17.6%)	2 (33.3%)	0 (0.0%)	5 (25.0%)	5 (13.5%)
Tinzaparin 4,500 units SubQ once daily	1 (6.7%)	0 (0.0%)	1 (4.0%)	1 (9.1%)	1 (2.9%)	0 (0.0%)	0 (0.0%)	2 (10.0%)	0 (0.0%)
Unfractionated heparin 5,000 units SubQ every 8 to 12 hours	12 (80.0%)	15 (88.2%)	19 (76.0%)	9 (81.8%)	29 (85.3%)	3 (50.0%)	5 (83.3%)	18 (90.0%)	28 (75.7%)

Table S2: Type pharmacological VTE PROPHYLAXIS used in patients with CKD at various stages (more than one option could be chosen)

Variable	YEARS EXPERIANCE			PRACTICE SETTING				SPECIALITY	
	5-10 years	< 5 years	> 10 years	Both	Government Hospital	Outpatient dialysis units ONLY	Private Hospital	Hematology track	Nephrology track
**[CKD stage 4 (eGFR 15-29)]**									
DOACs	8 (57.1%)	7 (41.2%)	10 (45.5%)	2 (22.2%)	13 (40.6%)	5 (83.3%)	5 (83.3%)	7 (38.9%)	18 (51.4%)
Fondaparinux	2 (14.3%)	0 (0.0%)	5 (22.7%)	2 (22.2%)	5 (15.6%)	0 (0.0%)	0 (0.0%)	4 (22.2%)	3 (8.6%)
Mechanical prophylaxis	12 (85.7%)	11 (64.7%)	9 (40.9%)	6 (66.7%)	20 (62.5%)	2 (33.3%)	4 (66.7%)	11 (61.1%)	21 (60.0%)
None	0 (0.0%)	1 (5.9%)	0 (0.0%)	0 (0.0%)	1 (3.1%)	0 (0.0%)	0 (0.0%)	0 (0.0%)	1 (2.9%)
Others	2 (14.3%)	1 (5.9%)	2 (9.1%)	0 (0.0%)	5 (15.6%)	0 (0.0%)	0 (0.0%)	0 (0.0%)	5 (14.3%)
Missing	1 (6.7%)	0 (0.0%)	3 (12.0%)	2 (18.2%)	2 (5.9%)	0 (0.0%)	0 (0.0%)	2 (10.0%)	2 (5.4%)
**[CKD stage 5 (eGFR <15)]**									
DOACs	5 (35.7%)	5 (31.2%)	9 (40.9%)	3 (33.3%)	10 (32.3%)	5 (83.3%)	5 (83.3%)	5 (29.4%)	18 (51.4%)
Fondaparinux	2 (14.3%)	2 (12.5%)	6 (27.3%)	1 (11.1%)	7 (22.6%)	0 (0.0%)	0 (0.0%)	4 (23.5%)	4 (11.4%)
Mechanical prophylaxis	12 (85.7%)	10 (62.5%)	9 (40.9%)	5 (55.6%)	19 (61.3%)	2 (33.3%)	4 (66.7%)	10 (58.8%)	20 (57.1%)
None	0 (0.0%)	1 (6.2%)	0 (0.0%)	0 (0.0%)	1 (3.2%)	0 (0.0%)	0 (0.0%)	0 (0.0%)	1 (2.9%)
Others	4 (28.6%)	2 (12.5%)	3 (13.6%)	1 (11.1%)	6 (19.4%)	0 (0.0%)	0 (0.0%)	2 (11.8%)	5 (14.3%)
Missing	1 (6.7%)	1 (5.9%)	3 (12.0%)	2 (18.2%)	3 (8.8%)	0 (0.0%)	0 (0.0%)	3 (15.0%)	2 (5.4%)
**[Dialysis]**									
DOACs	8 (53.3%)	9 (52.9%)	6 (24.0%)	2 (22.2%)	7 (22.6%)	5 (83.3%)	5 (83.3%)	4 (23.5%)	15 (42.9%)
Fondaparinux	1 (6.7%)	0 (0.0%)	4 (16.0%)	2 (22.2%)	8 (25.8%)	0 (0.0%)	0 (0.0%)	4 (23.5%)	6 (17.1%)
Mechanical prophylaxis	3 (20.0%)	4 (23.5%)	3 (12.0%)	6 (66.7%)	19 (61.3%)	2 (33.3%)	4 (66.7%)	11 (64.7%)	20 (57.1%)
None	5 (33.3%)	5 (29.4%)	7 (28.0%)	0 (0.0%)	1 (3.2%)	0 (0.0%)	0 (0.0%)	0 (0.0%)	1 (2.9%)
Others	8 (53.3%)	7 (41.2%)	8 (32.0%)	1 (11.1%)	8 (25.8%)	0 (0.0%)	0 (0.0%)	3 (15.0%)	6 (17.1%)
Missing	0 (0.0%)	0 (0.0%)	0 (0.0%)	2 (18.2%)	3 (8.8%)	0 (0.0%)	0 (0.0%)	3 (15.0%)	2 (5.4%)

Table S3: Anticoagulation agents alternative to heparin for VTE PROPHYLAXIS in patients with CKD at various stages

Variable	YEARS EXPERIANCE			PRACTICE SETTING				SPECIALITY	
	5-10 years	< 5 years	> 10 years	Both	Government Hospital	Outpatient dialysis units ONLY	Private Hospital	Hematology track	Nephrology track
[CKD stage 4 (eGFR 15-29)]									
DOACs	8 (53.3%)	9 (52.9%)	6 (24.0%)	2 (18.2%)	13 (38.2%)	4 (66.7%)	4 (66.7%)	12 (60.0%)	11 (29.7%)
I refer them to Hematologist or thrombosis expert	4 (26.7%)	4 (23.5%)	7 (28.0%)	3 (27.3%)	11 (32.3%)	0 (0.0%)	1 (16.7%)	1 (5.0%)	14 (37.8%)
LMWH	5 (33.3%)	5 (29.4%)	7 (28.0%)	2 (18.2%)	13 (38.2%)	1 (16.7%)	1 (16.7%)	7 (35.0%)	10 (27.0%)
Unfractionated heparin	8 (53.3%)	7 (41.2%)	8 (32.0%)	5 (45.5%)	14 (41.2%)	2 (33.3%)	2 (33.3%)	6 (30.0%)	17 (45.9%)
[CKD stage 5 (eGFR <15)]									
DOACs	5 (33.3%)	9 (52.9%)	3 (12.0%)	2 (18.2%)	8 (23.5%)	3 (50.0%)	4 (66.7%)	8 (40.0%)	9 (24.3%)
I refer them to Hematologist or thrombosis expert	6 (40 %)	4 (23.5%)	6 (24.0%)	3 (27.3%)	10 (29.4%)	1 (16.7%)	1 (16.7%)	2 (10 %)	14 (37.8%)
LMWH	4 (26.7%)	2 (11.8%)	5 (20.0%)	0 (0.0%)	10 (29.4%)	0 (0.0%)	1 (16.7%)	1 (5.0%)	10 (27.0%)
Unfractionated heparin	9 (60.0%)	9 (52.9%)	13 (52.0%)	7 (63.6%)	22 (64.7%)	2 (33.3%)	2 (33.3%)	14 (70.0%)	17 (45.9%)
[Dialysis]									
DOACs	5 (33.3%)	8 (47.1%)	4 (16.0%)	2 (18.2%)	8 (23.5%)	3 (50.0%)	4 (66.7%)	8 (40.0%)	9 (24.3%)
I refer them to Hematologist or thrombosis expert	6 (40 %)	3 (17.6%)	6 (24.0%)	3 (27.3%)	10 (29.4%)	1 (16.7%)	1 (16.7%)	2 (10 %)	13 (35.1%)
LMWH	4 (26.7%)	2 (11.8%)	5 (20.0%)	0 (0.0%)	10 (29.4%)	0 (0.0%)	1 (16.7%)	2 (10.0%)	9 (24.3%)
Unfractionated heparin	9 (60.0%)	8 (47.1%)	16 (64.0%)	7 (63.6%)	22 (64.7%)	2 (33.3%)	2 (33.3%)	13 (65.0%)	20 (54.1%)
[Kidney transplant]									
DOACs	5 (33.3%)	8 (47.1%)	6 (24.0%)	3 (27.3%)	11 (32.4%)	1 (16.7%)	4 (66.7%)	11 (55.0%)	8 (21.6%)
I refer them to Hematologist or thrombosis expert	5 (33.3%)	5 (29.4%)	6 (24.0%)	3 (27.3%)	11 (32.4%)	1 (16.7%)	1 (16.7%)	2 (10 %)	14 (37.8%)
LMWH	9 (60.0%)	5 (29.4%)	10 (40.0%)	3 (27.3%)	17 (50.0%)	2 (33.3%)	2 (33.3%)	8 (40.0%)	16 (43.2%)
Unfractionated heparin	5 (33.3%)	3 (17.6%)	10 (40.0%)	2 (18.2%)	12 (35.3%)	3 (50.0%)	1 (16.7%)	6 (30.0%)	12 (32.4%)

Table S4: Anticoagulation agents used for acute phase treatment of VTE

Variable	YEARS EXPERIANCE			PRACTICE SETTING				SPECIALITY	
	5-10 years	< 5 years	> 10 years	Both	Government Hospital	Outpatient dialysis units ONLY	Private Hospital	Hematology track	Nephrology track
[CKD stage 4 (eGFR 15-29)]									
Dalteparin (reduced dose)	1 (6.7%)	1 (5.9%)	0 (0.0%)	1 (9.1%)	0 (0.0%)	1 (16.7%)	0 (0.0%)	1 (5.0%)	1 (2.7%)
Enoxaparin (reduced dose)	9 (60.0%)	10 (58.8%)	18 (72.0%)	7 (63.6%)	23 (67.6%)	3 (50.0%)	4 (66.7%)	10 (50.0%)	27 (73.0%)
Enoxaparin (usual dose)	2 (13.3%)	1 (5.9%)	4 (16.0%)	2 (18.2%)	4 (11.8%)	1 (16.7%)	0 (0.0%)	2 (10.0%)	5 (13.5%)
I don't use LMWH	3 (20.0%)	5 (29.4%)	2 (8.0%)	0 (0.0%)	7 (20.6%)	1 (16.7%)	2 (33.3%)	6 (30.0%)	4 (10.8%)
Tinzaparin (usual dose)	1 (6.7%)	0 (0.0%)	1 (4.0%)	1 (9.1%)	0 (0.0%)	1 (16.7%)	0 (0.0%)	1 (5.0%)	1 (2.7%)
[CKD stage 5 (eGFR <15)]									
Dalteparin (reduced dose)	1 (6.7%)	1 (5.9%)	0 (0.0%)	1 (9.1%)	0 (0.0%)	1 (16.7%)	0 (0.0%)	0 (0.0%)	2 (5.4%)
Enoxaparin (reduced dose)	6 (40.0%)	7 (41.2%)	17 (68.0%)	6 (54.5%)	17 (50.0%)	3 (50.0%)	4 (66.7%)	6 (30.0%)	24 (64.9%)
Enoxaparin (usual dose)	1 (6.7%)	1 (5.9%)	3 (12.0%)	1 (9.1%)	3 (8.8%)	1 (16.7%)	0 (0.0%)	1 (5.0%)	4 (10.8%)
I don't use LMWH	6 (40.0%)	8 (47.1%)	5 (20.0%)	2 (18.2%)	14 (41.2%)	1 (16.7%)	2 (33.3%)	12 (60.0%)	7 (18.9%)
Tinzaparin (usual dose)	1 (6.7%)	0 (0.0%)	0 (0.0%)	1 (9.1%)	0 (0.0%)	0 (0.0%)	0 (0.0%)	1 (5.0%)	0 (0.0%)
[Dialysis]									

Dalteparin (reduced dose)	0 (0.0%)	1 (5.9%)	0 (0.0%)	1 (9.1%)	0 (0.0%)	0 (0.0%)	0 (0.0%)	0 (0.0%)	1 (2.7%)
Enoxaparin (reduced dose)	6 (40.0%)	6 (35.3%)	14 (56.0%)	5 (45.5%)	14 (41.2%)	3 (50.0%)	4 (66.7%)	3 (15.0%)	23 (62.2%)
Enoxaparin (usual dose)	1 (6.7%)	1 (5.9%)	5 (20.0%)	1 (9.1%)	5 (14.7%)	1 (16.7%)	0 (0.0%)	3 (15.0%)	4 (10.8%)
I don't use LMWH	7 (46.7%)	9 (52.9%)	5 (20.0%)	2 (18.2%)	15 (44.1%)	2 (33.3%)	2 (33.3%)	13 (65.0%)	8 (21.6%)
Tinzaparin (usual dose)	1 (6.7%)	1 (5.9%)	1 (4.0%)	2 (18.2%)	0 (0.0%)	0 (0.0%)	1 (16.7%)	1 (5.0%)	2 (5.4%)

Table S5: type and dose of LMWH for VTE treatment

Variable	YEARS EXPERIANCE			PRACTICE SETTING				SPECIALITY	
	**5-10 years**	**< 5 years**	**> 10 years**	**Both**	**Government Hospital**	**Outpatient dialysis units ONLY**	**Private Hospital**	**Hematology track**	**Nephrology track**
**[CKD stage 4 (eGFR 15-29)]**									
Both peak and trough anti-Xa	0 (0.0%)	1 (5.9%)	3 (12.0%)	2 (18.2%)	2 (5.9%)	0 (0.0%)	0 (0.0%)	2 (10.0%)	2 (5.4%)
I refer them to Hematologist or thrombosis expert	1 (6.7%)	2 (11.8%)	6 (24.0%)	3 (27.3%)	4 (11.8%)	1 (16.7%)	1 (16.7%)	0 (0.0%)	9 (24.3%)
Peak anti-Xa	1 (6.7%)	3 (17.6%)	5 (20.0%)	1 (9.1%)	5 (14.7%)	1 (16.7%)	2 (33.3%)	5 (25.0%)	4 (10.8%)
Trough anti-Xa	3 (20.0%)	4 (23.5%)	3 (12.0%)	3 (27.3%)	5 (14.7%)	1 (16.7%)	1 (16.7%)	6 (30.0%)	4 (10.8%)
Wouldn’t monitor	10 (66.7%)	7 (41.2%)	8 (32.0%)	2 (18.2%)	18 (52.9%)	3 (50.0%)	2 (33.3%)	7 (35.0%)	18 (48.6%)
**[CKD stage 5 (eGFR <15)]**									
Both peak and trough anti-Xa	1 (6.7%)	1 (5.9%)	4 (16.0%)	3 (27.3%)	2 (5.9%)	1 (16.7%)	0 (0.0%)	3 (15.0%)	3 (8.1%)
I refer them to Hematologist or thrombosis expert	1 (6.7%)	2 (11.8%)	8 (32.0%)	3 (27.3%)	6 (17.7%)	1 (16.7%)	1 (16.7%)	0 (0.0%)	11 (29.7%)
Peak anti-Xa	1 (6.7%)	3 (17.6%)	5 (20.0%)	1 (9.1%)	5 (14.7%)	1 (16.7%)	2 (33.3%)	6 (30.0%)	3 (8.1%)
Trough anti-Xa	3 (20.0%)	4 (23.5%)	3 (12.0%)	3 (27.3%)	5 (14.7%)	1 (16.7%)	1 (16.7%)	5 (25.0%)	5 (13.5%)
Wouldn’t monitor	9 (60.0%)	7 (41.2%)	5 (20.0%)	1 (9.1%)	16 (47.1%)	2 (33.3%)	2 (33.3%)	6 (30.0%)	15 (40.5%)
**[Dialysis]**									
Both peak and trough anti-Xa	0 (0.0%)	1 (5.9%)	5 (20.0%)	4 (36.4%)	2 (5.9%)	0 (0.0%)	0 (0.0%)	3 (15.0%)	3 (8.1%)
I refer them to Hematologist or thrombosis expert	3 (20.0%)	2 (11.8%)	7 (28.0%)	3 (27.3%)	6 (17.7%)	2 (33.3%)	1 (16.7%)	0 (0.0%)	12 (32.4%)
Peak anti-Xa	1 (6.7%)	3 (17.6%)	4 (16.0%)	1 (9.1%)	4 (11.8%)	1 (16.7%)	2 (33.3%)	5 (25.0%)	3 (8.1%)
Trough anti-Xa	2 (13.3%)	4 (23.5%)	1 (4.0%)	2 (18.2%)	3 (8.8%)	1 (16.7%)	1 (16.7%)	4 (20.0%)	3 (8.1%)
Wouldn’t monitor	9 (60.0%)	7 (41.2%)	8 (32.0%)	1 (9.1%)	19 (55.9%)	2 (33.3%)	2 (33.3%)	8 (40.0%)	16 (43.2%)

Table S6: Monitoring of LMWH during VTE treatment

Variable	YEARS EXPERIANCE			PRACTICE SETTING				SPECIALITY	
	5-10 years	< 5 years	> 10 years	Both	Government Hospital	Outpatient dialysis units ONLY	Private Hospital	Hematology track	Nephrology track
**[CKD stage 4 (eGFR 15-29)]**									
DOAC	11 (73.3%)	10 (58.8%)	10 (40.0%)	4 (36.4%)	20 (58.8%)	2 (33.3%)	5 (83.3%)	13 (65.0%)	18 (48.6%)
I refer them to Hematologist or thrombosis expert	5 (33.4%)	2 (11.8%)	7 (28.0%)	2 (18.2%)	10 (29.4%)	1 (16.7%)	1 (16.7%)	1 (5.0%)	13 (35.1%)
LMWH	1 (6.7%)	3 (17.6%)	5 (20.0%)	2 (18.2%)	6 (17.6%)	1 (16.7%)	0 (0.0%)	4 (20.0%)	5 (13.5%)
VKA (warfarin)	7 (46.7%)	9 (52.9%)	8 (32.0%)	4 (36.4%)	14 (41.2%)	4 (66.7%)	2 (33.3%)	8 (40.0%)	16 (43.2%)
**[CKD stage 5 (eGFR <15)]**									
DOAC	8 (53.3%)	8 (47.1%)	6 (24.0%)	3 (27.3%)	12 (35.3%)	3 (50.0%)	4 (66.7%)	6 (30.0%)	16 (43.2%)
I refer them to Hematologist or thrombosis expert	6 (40.0%)	2 (11.8%)	8 (32.0%)	2 (18.2%)	12 (35.3%)	1 (16.7%)	1 (16.7%)	2 (10.0%)	14 (37.8%)
LMWH	1 (6.7%)	2 (11.8%)	2 (8.0%)	0 (0.0%)	4 (11.8%)	1 (16.7%)	0 (0.0%)	2 (10.0%)	3 (8.1%)
VKA (warfarin)	8 (53.3%)	10 (58.8%)	13 (52.0%)	7 (63.6%)	18 (52.9%)	3 (50.0%)	3 (50.0%)	14 (70.0%)	17 (45.9%)
**[Dialysis]**									
DOAC	8 (53.3%)	8 (47.1%)	6 (24.0%)	3 (27.3%)	12 (35.3%)	3 (50.0%)	4 (66.7%)	6 (30.0%)	16 (43.2%)
I refer them to Hematologist or thrombosis expert	6 (40.0%)	2 (11.8%)	7 (28.0%)	2 (18.2%)	11 (32.4%)	1 (16.7%)	1 (16.7%)	2 (10.0%)	13 (35.1%)
LMWH	2 (13.3%)	2 (11.8%)	3 (12.0%)	0 (0.0%)	5 (14.7%)	2 (33.3%)	0 (0.0%)	2 (10.0%)	5 (13.5%)
VKA (warfarin)	8 (53.3%)	9 (52.9%)	13 (52.0%)	7 (63.6%)	18 (52.9%)	2 (33.3%)	3 (50.0%)	13 (65.0%)	17 (45.9%)
**[Kidney transplant]**									
DOAC	12 (80.0%)	12 (70.6%)	7 (28.0%)	5 (45.5%)	20 (58.8%)	2 (33.3%)	4 (66.7%)	14 (70.0%)	17 (45.9%)
I refer them to Hematologist or thrombosis expert	7 (46.7%)	3 (17.6%)	8 (32.0%)	2 (18.2%)	12 (35.3%)	3 (40.0%)	1 (16.7%)	2 (10.0%)	16 (43.2%)
LMWH	3 (20.0%)	4 (23.5%)	5 (20.0%)	3 (27.3%)	8 (23.5%)	0 (0.0%)	1 (16.7%)	4 (20.0%)	8 (21.6%)
VKA (warfarin)	3 (20.0%)	4 (23.5%)	10 (40.0%)	3 (27.3%)	11 (32.4%)	1 (16.7%)	2 (33.3%)	7 (35.0%)	10 (27.0%)

Table S7: Anticoagulation agents used for chronic phase treatment of VTE

Variable	YEARS EXPERIANCE			PRACTICE SETTING				SPECIALI[illegible]	
	5-10 years	< 5 years	> 10 years	Both	Government Hospital	Outpatient dialysis units ONLY	Private Hospital	Hematology track	N[illegible]
**[CKD stage 4 (eGFR 15-29)]**									
Apixaban 2.5 mg twice a day	12 (80.0%)	10 (58.8%)	11 (44.0%)	6 (54.5%)	21 (61.8%)	2 (33.3%)	4 (66.7%)	11 (55.0%)	22 [illegible]
Apixaban 5 mg twice a day	4 (26.7%)	5 (29.4%)	9 (36.0%)	2 (18.2%)	11 (32.4%)	3 (50.0%)	2 (33.3%)	8 (40.0%)	10 [illegible]
Dabigatran 150 mg twice a day	0 (0.0%)	0 (0.0%)	1 (4.0%)	0 (0.0%)	0 (0.0%)	1 (16.7%)	0 (0.0%)	0 (0.0%)	1 [illegible]
Dabigatran 75 mg twice a day	0 (0.0%)	1 (5.9%)	1 (4.0%)	1 (9.1%)	1 (2.9%)	0 (0.0%)	0 (0.0%)	1 (5.0%)	1 [illegible]
I don't use DOAC	0 (0.0%)	2 (11.8%)	4 (16.0%)	2 (18.2%)	3 (8.8%)	0 (0.0%)	1 (16.7%)	3 (15.0%)	3 [illegible]
Rivaroxaban 10 mg daily	2 (13.3%)	2 (11.8%)	1 (4.0%)	1 (9.1%)	4 (11.8%)	0 (0.0%)	0 (0.0%)	2 (10.0%)	3 [illegible]
Rivaroxaban 15 mg daily	2 (13.3%)	0 (0.0%)	2 (8.0%)	0 (0.0%)	4 (11.8%)	0 (0.0%)	0 (0.0%)	2 (10.0%)	2 [illegible]
**[CKD stage 5 (eGFR <15)]**									
Apixaban 2.5 mg twice a day	11 (73.3%)	12 (70.6%)	14 (56.0%)	7 (63.6%)	21 (61.8%)	4 (66.7%)	5 (83.3%)	8 (40.0%)	29 [illegible]
Apixaban 5 mg twice a day	3 (20.0%)	2 (11.8%)	6 (24.0%)	2 (18.2%)	7 (20.6%)	1 (16.7%)	1 (16.7%)	8 (40.0%)	3 [illegible]
Dabigatran 150 mg twice a day	0 (0.0%)	0 (0.0%)	2 (8.0%)	1 (9.1%)	0 (0.0%)	1 (16.7%)	0 (0.0%)	0 (0.0%)	2 [illegible]
Dabigatran 75 mg twice a day	0 (0.0%)	1 (5.9%)	0 (0.0%)	0 (0.0%)	1 (2.9%)	0 (0.0%)	0 (0.0%)	1 (5.0%)	0 [illegible]
I don't use DOAC	2 (13.3%)	3 (17.6%)	5 (20.0%)	2 (18.2%)	7 (20.6%)	0 (0.0%)	1 (16.7%)	7 (35.0%)	3 [illegible]
Rivaroxaban 10 mg daily	2 (13.3%)	0 (0.0%)	0 (0.0%)	0 (0.0%)	2 (5.9%)	0 (0.0%)	0 (0.0%)	0 (0.0%)	2 [illegible]
Rivaroxaban 15 mg daily	1 (6.7%)	0 (0.0%)	1 (4.0%)	0 (0.0%)	2 (5.9%)	0 (0.0%)	0 (0.0%)	1 (5.0%)	1 [illegible]
**[Dialysis]**									
Apixaban 2.5 mg twice a day	9 (60.0%)	10 (58.8%)	11 (44.0%)	5 (45.5%)	18 (52.9%)	2 (33.3%)	5 (83.3%)	5 (25.0%)	25 [illegible]
Apixaban 5 mg twice a day	2 (13.3%)	2 (11.8%)	6 (24.0%)	1 (9.1%)	6 (17.6%)	2 (33.3%)	1 (16.7%)	5 (25.0%)	5 [illegible]
Dabigatran 150 mg twice a day	0 (0.0%)	0 (0.0%)	2 (8.0%)	1 (9.1%)	0 (0.0%)	1 (16.7%)	0 (0.0%)	0 (0.0%)	2 [illegible]
Dabigatran 75 mg twice a day	5 (33.3%)	5 (29.4%)	6 (24.0%)	3 (27.3%)	11 (32.4%)	1 (16.7%)	1 (16.7%)	11 (55.0%)	5 [illegible]
I don't use DOAC	2 (13.3%)	0 (0.0%)	0 (0.0%)	0 (0.0%)	2 (5.9%)	0 (0.0%)	0 (0.0%)	0 (0.0%)	2 [illegible]
Rivaroxaban 10 mg daily	1 (6.7%)	0 (0.0%)	1 (4.0%)	0 (0.0%)	2 (5.9%)	0 (0.0%)	0 (0.0%)	1 (5.0%)	1 [illegible]
Rivaroxaban 15 mg daily	0 (0.0%)	0 (0.0%)	1 (4.0%)	1 (9.1%)	0 (0.0%)	0 (0.0%)	0 (0.0%)	1 (5.0%)	0 [illegible]

Table S8: DOACs for treatment of VTE which DOAC and what dose would you use

Variable	YEARS EXPERIANCE		PRACTICE SETTING				SPECIALITY		
	5-10 years	< 5 years	> 10 years	Both	Government Hospital	Outpatient dialysis units ONLY	Private Hospital	Hematology track	Nephrology track
According to hematologist opinion	1 (6.7%)	0 (0.0%)	0 (0.0%)	0 (0.0%)	1 (2.9%)	0 (0.0%)	0 (0.0%)	0 (0.0%)	1 (2.7%)
Both	10 (66.7%)	10 (58.8%)	9 (36.0%)	9 (81.8%)	15 (44.1%)	2 (33.3%)	3 (50.0%)	12 (60.0%)	17 (45.9%)
CHADSVASc	3 (20.0%)	7 (41.2%)	9 (36.0%)	2 (18.2%)	11 (32.4%)	3 (50.0%)	3 (50.0%)	6 (30.0%)	13 (35.1%)
Don’t use risk scores	1 (6.7%)	0 (0.0%)	6 (24.0%)	0 (0.0%)	6 (17.6%)	1 (16.7%)	0 (0.0%)	2 (10.0%)	5 (13.5%)
Hematology referral to decide	0 (0.0%)	0 (0.0%)	1 (4.0%)	0 (0.0%)	1 (2.9%)	0 (0.0%)	0 (0.0%)	0 (0.0%)	1 (2.7%)

TableS9: Atrial fibrillation related risk scores routinely used in decision making when anticoagulating CKD patients with AF

Variable	YEARS EXPERIANCE			PRACTICE SETTING				SPECIALITY	
	5-10 years	< 5 years	> 10 years	Both	Government Hospital	Outpatient dialysis units ONLY	Private Hospital	Hematology track	Nephrology track
**[CKD stage 4 (eGFR 15-29)]**									
Discuss at multi-disciplinary team (MDT) round	1 (6.7%)	1 (5.9%)	8 (32.0%)	2 (18.2%)	4 (11.8%)	2 (33.3%)	2 (33.3%)	2 (10.0%)	8 (21.6%)
Individual decision made with patient	3 (20.0%)	6 (35.3%)	2 (8.0%)	2 (18.2%)	8 (23.5%)	1 (16.7%)	0 (0.0%)	2 (10.0%)	9 (24.3%)
No	0 (0.0%)	0 (0.0%)	2 (8.0%)	1 (9.1%)	0 (0.0%)	1 (16.7%)	0 (0.0%)	0 (0.0%)	2 (5.4%)
Not sure	1 (6.7%)	1 (5.9%)	1 (4.0%)	0 (0.0%)	3 (8.8%)	0 (0.0%)	0 (0.0%)	2 (10.0%)	1 (2.7%)
Yes	10 (66.7%)	9 (52.9%)	12 (48.0%)	6 (54.5%)	19 (55.9%)	2 (33.3%)	4 (66.7%)	14 (70.0%)	17 (45.9%)
**[CKD stage 5 (eGFR <15)]**									
Discuss at multi-disciplinary team (MDT) round	3 (20.0%)	1 (5.9%)	7 (28.0%)	1 (9.1%)	6 (17.6%)	2 (33.3%)	2 (33.3%)	2 (10.0%)	9 (24.3%)
Individual decision made with patient	2 (13.3%)	6 (35.3%)	4 (16.0%)	2 (18.2%)	9 (26.5%)	1 (16.7%)	0 (0.0%)	3 (15.0%)	9 (24.3%)
No	0 (0.0%)	0 (0.0%)	5 (20.0%)	3 (27.3%)	2 (5.9%)	0 (0.0%)	0 (0.0%)	2 (10.0%)	3 (8.1%)
Not sure	1 (6.7%)	1 (5.9%)	2 (8.0%)	0 (0.0%)	4 (11.8%)	0 (0.0%)	0 (0.0%)	2 (10.0%)	2 (5.4%)
Yes	9 (60.0%)	9 (52.9%)	7 (28.0%)	5 (45.5%)	13 (38.2%)	3 (50.0%)	4 (66.7%)	11 (55.0%)	14 (37.8%)
**[Dialysis]**									
Discuss at multi-disciplinary team (MDT) round	2 (13.3%)	1 (5.9%)	11 (44.0%)	2 (18.2%)	7 (20.6%)	3 (50.0%)	2 (33.3%)	2 (10.0%)	12 (32.4%)
Individual decision made with patient	4 (26.7%)	6 (35.3%)	3 (12.0%)	1 (9.1%)	10 (29.4%)	2 (33.3%)	0 (0.0%)	3 (15.0%)	10 (27.0%)
No	1 (6.7%)	0 (0.0%)	3 (12.0%)	2 (18.2%)	2 (5.9%)	0 (0.0%)	0 (0.0%)	2 (10.0%)	2 (5.4%)
Not sure	1 (6.7%)	2 (11.8%)	3 (12.0%)	1 (9.1%)	5 (14.7%)	0 (0.0%)	0 (0.0%)	2 (10.0%)	4 (10.8%)
Yes	7 (46.7%)	8 (47.1%)	5 (20.0%)	5 (45.5%)	10 (29.4%)	1 (16.7%)	4 (66.7%)	11 (55.0%)	9 (24.3%)

Table S10: Decision to anticoagulate patients with Afib and CKD who have CHADSVASC > 2 (moderate thrombosis risk) with HASBLED < 3 (low bleeding risk)

Variable	YEARS EXPERIANCE			PRACTICE SETTING				SPECIALITY	
	5-10 years	< 5 years	> 10 years	Both	Government Hospital	Outpatient dialysis units ONLY	Private Hospital	Hematology track	Nephrology track
**[CKD stage 4 (eGFR 15-29)]**									
Discuss at multi-disciplinary team (MDT) round	3 (21.4%)	4 (23.5%)	5 (20.0%)	1 (9.1%)	5 (15.2%)	3 (50.0%)	3 (50.0%)	2 (10.0%)	10 (27.8%)
Individual decision made with patient	4 (28.6%)	7 (41.2%)	8 (32.0%)	3 (27.3%)	12 (36.4%)	2 (33.3%)	2 (33.3%)	6 (30.0%)	13 (36.1%)
No	3 (21.4%)	1 (5.9%)	2 (8.0%)	3 (27.3%)	2 (6.1%)	0 (0.0%)	1 (16.7%)	2 (10.0%)	4 (11.1%)
Not sure	2 (14.3%)	3 (17.6%)	3 (12.0%)	2 (18.2%)	5 (15.2%)	1 (16.7%)	0 (0.0%)	6 (30.0%)	2 (5.6%)
Yes	2 (14.3%)	2 (11.8%)	7 (28.0%)	2 (18.2%)	9 (27.3%)	0 (0.0%)	0 (0.0%)	4 (20.0%)	7 (19.4%)
Missing	1 (6.7%)	0 (0.0%)	0 (0.0%)	0 (0.0%)	1 (2.9%)	0 (0.0%)	0 (0.0%)	0 (0.0%)	1 (2.7%)
**[CKD stage 5 (eGFR <15)]**									
Discuss at multi-disciplinary team (MDT) round	4 (28.6%)	4 (23.5%)	5 (20.0%)	1 (9.1%)	5 (15.2%)	4 (66.7%)	3 (50.0%)	2 (10.0%)	11 (30.6%)
Individual decision made with patient	3 (21.4%)	8 (47.1%)	7 (28.0%)	4 (36.4%)	10 (30.3%)	2 (33.3%)	2 (33.3%)	6 (30.0%)	12 (33.3%)
No	4 (28.6%)	1 (5.9%)	6 (24.0%)	4 (36.4%)	6 (18.2%)	0 (0.0%)	1 (16.7%)	3 (15.0%)	8 (22.2%)
Not sure	1 (7.1%)	3 (17.6%)	5 (20.0%)	2 (18.2%)	7 (21.2%)	0 (0.0%)	0 (0.0%)	7 (35.0%)	2 (5.6%)
Yes	2 (14.3%)	1 (5.9%)	2 (8.0%)	0 (0.0%)	5 (15.2%)	0 (0.0%)	0 (0.0%)	2 (10.0%)	3 (8.3%)
Missing	1 (6.7%)	0 (0.0%)	0 (0.0%)	0 (0.0%)	1 (2.9%)	0 (0.0%)	0 (0.0%)	0 (0.0%)	1 (2.7%)
**[Dialysis]**									
Discuss at multi-disciplinary team (MDT) round	2 (14.3%)	4 (23.5%)	6 (24.0%)	2 (18.2%)	4 (12.1%)	3 (50.0%)	3 (50.0%)	2 (10.0%)	10 (27.8%)
Individual decision made with patient	4 (28.6%)	8 (47.1%)	6 (24.0%)	3 (27.3%)	10 (30.3%)	3 (50.0%)	2 (33.3%)	6 (30.0%)	12 (33.3%)
No	5 (35.7%)	1 (5.9%)	7 (28.0%)	4 (36.4%)	8 (24.2%)	0 (0.0%)	1 (16.7%)	5 (25.0%)	8 (22.2%)
Not sure	1 (7.1%)	3 (17.6%)	4 (16.0%)	2 (18.2%)	6 (18.2%)	0 (0.0%)	0 (0.0%)	5 (25.0%)	3 (8.3%)
Yes	2 (14.3%)	1 (5.9%)	2 (8.0%)	0 (0.0%)	5 (15.2%)	0 (0.0%)	0 (0.0%)	2 (10.0%)	3 (8.3%)
Missing	1 (6.7%)	0 (0.0%)	0 (0.0%)	0 (0.0%)	1 (2.9%)	0 (0.0%)	0 (0.0%)	0 (0.0%)	1 (2.7%)

Table S11: Decision to anticoagulate patients with Afib and CKD who have CHADSVASC>2 (moderate thrombosis risk) with HASBLED>3 (high bleeding risk)

Variable	YEARS EXPERIANCE			PRACTICE SETTING				SPECIALITY	
	5-10 years	< 5 years	> 10 years	Both	Government Hospital	Outpatient dialysis units ONLY	Private Hospital	Hematology track	Nephrology track
**[CKD stage 4 (eGFR 15-29)]**									
Discuss at multi-disciplinary team (MDT) round	8 (53.3%)	3 (17.6%)	10 (41.7%)	2 (18.2%)	12 (35.3%)	4 (66.7%)	3 (60.0%)	5 (25.0%)	16 (44.4%)
Individual decision made with patient	3 (20.0%)	7 (41.2%)	3 (12.5%)	4 (36.4%)	8 (23.5%)	0 (0.0%)	1 (20.0%)	3 (15.0%)	10 (27.8%)
No	0 (0.0%)	2 (11.8%)	4 (16.7%)	2 (18.2%)	3 (8.8%)	1 (16.7%)	0 (0.0%)	2 (10.0%)	4 (11.1%)
Not sure	1 (6.7%)	0 (0.0%)	1 (4.2%)	0 (0.0%)	2 (5.9%)	0 (0.0%)	0 (0.0%)	1 (5.0%)	1 (2.8%)
Yes	3 (20.0%)	5 (29.4%)	6 (25.0%)	3 (27.3%)	9 (26.5%)	1 (16.7%)	1 (20.0%)	9 (45.0%)	5 (13.9%)
Missing	0 (0.0%)	0 (0.0%)	1 (4.0%)	0 (0.0%)	0 (0.0%)	0 (0.0%)	1 (16.7%)	0 (0.0%)	1 (2.7%)
**[CKD stage 5 (eGFR <15)]**									
Discuss at multi-disciplinary team (MDT) round	8 (53.3%)	3 (18.8%)	12 (48.0%)	2 (18.2%)	12 (36.4%)	6 (100.0%)	3 (50.0%)	6 (31.6%)	17 (45.9%)
Individual decision made with patient	4 (26.7%)	7 (43.8%)	3 (12.0%)	4 (36.4%)	8 (24.2%)	0 (0.0%)	2 (33.3%)	3 (15.8%)	11 (29.7%)
No	0 (0.0%)	2 (12.5%)	5 (20.0%)	3 (27.3%)	4 (12.1%)	0 (0.0%)	0 (0.0%)	2 (10.5%)	5 (13.5%)
Not sure	1 (6.7%)	1 (6.2%)	1 (4.0%)	0 (0.0%)	3 (9.1%)	0 (0.0%)	0 (0.0%)	2 (10.5%)	1 (2.7%)
Yes	2 (13.3%)	3 (18.8%)	4 (16.0%)	2 (18.2%)	6 (18.2%)	0 (0.0%)	1 (16.7%)	6 (31.6%)	3 (8.1%)
Missing	0 (0.0%)	1 (5.9%)	0 (0.0%)	0 (0.0%)	1 (2.9%)	0 (0.0%)	0 (0.0%)	1 (5.0%)	0 (0.0%)
**[Dialysis]**									
Discuss at multi-disciplinary team (MDT) round	8 (53.3%)	3 (17.6%)	13 (52.0%)	3 (27.3%)	13 (38.2%)	5 (83.3%)	3 (50.0%)	7 (35.0%)	17 (45.9%)
Individual decision made with patient	3 (20.0%)	8 (47.1%)	3 (12.0%)	4 (36.4%)	7 (20.6%)	1 (16.7%)	2 (33.3%)	3 (15.0%)	11 (29.7%)
No	1 (6.7%)	2 (11.8%)	4 (16.0%)	2 (18.2%)	5 (14.7%)	0 (0.0%)	0 (0.0%)	2 (10.0%)	5 (13.5%)
Not sure	1 (6.7%)	1 (5.9%)	2 (8.0%)	1 (9.1%)	3 (8.8%)	0 (0.0%)	0 (0.0%)	2 (10.0%)	2 (5.4%)
Yes	2 (13.3%)	3 (17.6%)	3 (12.0%)	1 (9.1%)	6 (17.6%)	0 (0.0%)	1 (16.7%)	6 (30.0%)	2 (5.4%)
Missing	0 (0.0%)	0 (0.0%)	0 (0.0%)	0 (0.0%)	0 (0.0%)	0 (0.0%)	0 (0.0%)	0 (0.0%)	0 (0.0%)

Table S12: Decision to anticoagulate patients with Afib and CKD who have anticoagulate CHADSVASC>5 (high thrombosis risk) with HASBLED>3 (high bleeding risk)

Variable	YEARS EXPERIANCE			PRACTICE SETTING				SPECIALITY	
	5-10 years	< 5 years	> 10 years	Both	Government Hospital	Outpatient dialysis units ONLY	Private Hospital	Hematology track	Nephrology track
**[CKD stage 4 (eGFR 15-29)]**									
DOACs	12 (80.0%)	11 (68.8%)	11 (44.0%)	5 (45.5%)	20 (60.6%)	4 (66.7%)	5 (83.3%)	13 (68.4%)	21 (56.8%)
I refer them to Hematologist (or thrombosis expert) or Cardiology	4 (26.7%)	4 (25.0%)	6 (24.0%)	2 (18.2%)	11 (33.3%)	0 (0.0%)	1 (16.7%)	1 (5.3%)	13 (35.1%)
VKA (warfarin)	5 (33.3%)	7 (43.8%)	11 (44.0%)	7 (63.6%)	11 (33.3%)	3 (50.0%)	2 (33.3%)	8 (42.1%)	15 (40.5%)
Missing	0 (0.0%)	1 (5.9%)	0 (0.0%)	0 (0.0%)	1 (2.9%)	0 (0.0%)	0 (0.0%)	1 (5.0%)	0 (0.0%)
**[CKD stage 5 (eGFR <15)]**									
DOACs	9 (60.0%)	9 (56.2%)	6 (24.0%)	4 (36.4%)	12 (36.4%)	3 (50.0%)	5 (83.3%)	6 (31.6%)	18 (48.6%)
I refer them to Hematologist (or thrombosis expert) or Cardiology	4 (26.7%)	4 (25.0%)	7 (28.0%)	3 (27.3%)	11 (33.3%)	0 (0.0%)	1 (16.7%)	2 (10.5%)	13 (35.1%)
VKA (warfarin)	6 (40.0%)	8 (50.0%)	14 (56.0%)	6 (54.5%)	17 (51.5%)	3 (50.0%)	2 (33.3%)	12 (63.2%)	16 (43.2%)
Missing	0 (0.0%)	1 (5.9%)	0 (0.0%)	0 (0.0%)	1 (2.9%)	0 (0.0%)	0 (0.0%)	1 (5.0%)	0 (0.0%)
**[Dialysis]**									
DOACs	8 (53.3%)	9 (56.2%)	9 (36.0%)	6 (54.5%)	13 (39.4%)	3 (50.0%)	4 (66.7%)	7 (36.8%)	19 (51.4%)
I refer them to Hematologist (or thrombosis expert) or Cardiology	5 (33.3%)	4 (25.0%)	6 (24.0%)	2 (18.2%)	11 (33.3%)	1 (16.7%)	1 (16.7%)	1 (5.3%)	14 (37.8%)
VKA (warfarin)	6 (40.0%)	8 (50.0%)	14 (56.0%)	5 (45.5%)	18 (54.5%)	2 (33.3%)	3 (50.0%)	13 (68.4%)	15 (40.5%)
Missing	0 (0.0%)	1 (5.9%)	0 (0.0%)	0 (0.0%)	1 (2.9%)	0 (0.0%)	0 (0.0%)	1 (5.0%)	0 (0.0%)
**[Kidney transplant]**									
DOACs	10 (66.7%)	10 (62.5%)	9 (36.0%)	6 (54.5%)	19 (57.6%)	1 (16.7%)	3 (50.0%)	15 (78.9%)	14 (37.8%)
I refer them to Hematologist (or thrombosis expert) or Cardiology	5 (33.3%)	4 (25.0%)	7 (28.0%)	2 (18.2%)	12 (36.4%)	1 (16.7%)	1 (16.7%)	1 (5.3%)	15 (40.5%)
VKA (warfarin)	4 (26.7%)	6 (37.5%)	14 (56.0%)	6 (54.5%)	11 (33.3%)	4 (66.7%)	3 (50.0%)	8 (42.1%)	16 (43.2%)
Missing	0 (0.0%)	1 (5.9%)	0 (0.0%)	0 (0.0%)	1 (2.9%)	0 (0.0%)	0 (0.0%)	1 (5.0%)	0 (0.0%)

Table S13: anticoagulants agents used in patients with AF and CKD (more than one option could be chosen)

Variable	YEARS EXPERIANCE			PRACTICE SETTING				SPECIALITY	
	5-10 years	< 5 years	> 10 years	Both	Government Hospital	Outpatient dialysis units ONLY	Private Hospital	Hematology track	Nephrology track
**[CKD stage 4 (eGFR 15-29)]**									
Apixaban 2.5 mg twice a day	14 (93.3%)	12 (70.6%)	14 (56.0%)	8 (72.7%)	22 (64.7%)	5 (83.3%)	5 (83.3%)	12 (60.0%)	28 (75.7%)
Apixaban 5 mg twice a day	3 (20.0%)	5 (29.4%)	9 (36.0%)	1 (9.1%)	13 (38.2%)	2 (33.3%)	1 (16.7%)	8 (40.0%)	9 (24.3%)
Dabigatran 75 mg twice a day	0 (0.0%)	1 (5.9%)	0 (0.0%)	0 (0.0%)	1 (2.9%)	0 (0.0%)	0 (0.0%)	1 (5.0%)	0 (0.0%)
I don't use DOAC	0 (0.0%)	1 (5.9%)	3 (12.0%)	2 (18.2%)	1 (2.9%)	0 (0.0%)	1 (16.7%)	2 (10.0%)	2 (5.4%)
Rivaroxaban 10 mg daily	3 (20.0%)	2 (11.8%)	0 (0.0%)	1 (9.1%)	3 (8.8%)	1 (16.7%)	0 (0.0%)	1 (5.0%)	4 (10.8%)
Rivaroxaban 15 mg daily	0 (0.0%)	0 (0.0%)	1 (4.0%)	0 (0.0%)	1 (2.9%)	0 (0.0%)	0 (0.0%)	1 (5.0%)	0 (0.0%)
**[CKD stage 5 (eGFR <15)]**									
Apixaban 2.5 mg twice a day	12 (80.0%)	12 (70.6%)	20 (80.0%)	8 (72.7%)	26 (76.5%)	5 (83.3%)	5 (83.3%)	11 (55.0%)	33 (89.2%)
Apixaban 5 mg twice a day	2 (13.3%)	2 (11.8%)	2 (8.0%)	0 (0.0%)	4 (11.8%)	1 (16.7%)	1 (16.7%)	4 (20.0%)	2 (5.4%)
Dabigatran 75 mg twice a day	0 (0.0%)	2 (11.8%)	0 (0.0%)	0 (0.0%)	2 (5.9%)	0 (0.0%)	0 (0.0%)	2 (10.0%)	0 (0.0%)
I don't use DOAC	2 (13.3%)	2 (11.8%)	4 (16.0%)	3 (27.3%)	4 (11.8%)	0 (0.0%)	1 (16.7%)	6 (30.0%)	2 (5.4%)
Rivaroxaban 10 mg daily	3 (20.0%)	0 (0.0%)	0 (0.0%)	0 (0.0%)	2 (5.9%)	1 (16.7%)	0 (0.0%)	0 (0.0%)	3 (8.1%)
Rivaroxaban 15 mg daily	0 (0.0%)	0 (0.0%)	1 (4.0%)	0 (0.0%)	1 (2.9%)	0 (0.0%)	0 (0.0%)	1 (5.0%)	0 (0.0%)
**[Dialysis]**									
Apixaban 2.5 mg twice a day	10 (66.7%)	12 (70.6%)	15 (60.0%)	6 (54.5%)	23 (67.6%)	3 (50.0%)	5 (83.3%)	8 (40.0%)	29 (78.4%)
Apixaban 5 mg twice a day	2 (13.3%)	2 (11.8%)	8 (32.0%)	2 (18.2%)	7 (20.6%)	2 (33.3%)	1 (16.7%)	8 (40.0%)	4 (10.8%)
Dabigatran 75 mg twice a day	0 (0.0%)	1 (5.9%)	0 (0.0%)	0 (0.0%)	1 (2.9%)	0 (0.0%)	0 (0.0%)	1 (5.0%)	0 (0.0%)
I don't use DOAC	4 (26.7%)	2 (11.8%)	3 (12.0%)	3 (27.3%)	4 (11.8%)	1 (16.7%)	1 (16.7%)	5 (25.0%)	4 (10.8%)
Rivaroxaban 10 mg daily	2 (13.3%)	0 (0.0%)	0 (0.0%)	0 (0.0%)	2 (5.9%)	0 (0.0%)	0 (0.0%)	0 (0.0%)	2 (5.4%)
Rivaroxaban 15 mg daily	0 (0.0%)	0 (0.0%)	1 (4.0%)	0 (0.0%)	1 (2.9%)	0 (0.0%)	0 (0.0%)	1 (5.0%)	0 (0.0%)

Table S14: type and dose of DOACs used in patients with AF and CKD (more than one option could be chosen)

21 Part b. Would you check or monitor DOAC levels (regardless of the ability of lab to perform such a test) ?									
Variable	YEARS EXPERIANCE			PRACTICE SETTING				SPECIALITY	
	5-10 years	< 5 years	> 10 years	Both	Government Hospital	Outpatient dialysis units ONLY	Private Hospital	Hematology track	Nephrology track
**[CKD stage 4 (eGFR 15-29)]**									
I refer them to Hematology	0 (0.0%)	3 (17.6%)	3 (12.0%)	2 (18.2%)	3 (8.8%)	0 (0.0%)	1 (16.7%)	0 (0.0%)	6 (16.2%)
No	11 (73.3%)	13 (76.5%)	11 (44.0%)	4 (36.4%)	23 (67.6%)	5 (83.3%)	3 (50.0%)	12 (60.0%)	23 (62.2%)
Yes- All patients	0 (0.0%)	0 (0.0%)	4 (16.0%)	1 (9.1%)	3 (8.8%)	0 (0.0%)	0 (0.0%)	3 (15.0%)	1 (2.7%)
Yes-In some patients	4 (26.7%)	1 (5.9%)	7 (28.0%)	4 (36.4%)	5 (14.7%)	1 (16.7%)	2 (33.3%)	5 (25.0%)	7 (18.9%)
**[CKD stage 5 (eGFR <15)]**									
I refer them to Hematology	1 (6.7%)	3 (17.6%)	3 (12.0%)	2 (18.2%)	4 (11.8%)	0 (0.0%)	1 (16.7%)	0 (0.0%)	7 (18.9%)
No	10 (66.7%)	13 (76.5%)	9 (36.0%)	4 (36.4%)	20 (58.8%)	5 (83.3%)	3 (50.0%)	10 (50.0%)	22 (59.5%)
Yes- All patients	0 (0.0%)	0 (0.0%)	6 (24.0%)	1 (9.1%)	5 (14.7%)	0 (0.0%)	0 (0.0%)	4 (20.0%)	2 (5.4%)
Yes-In some patients	4 (26.7%)	1 (5.9%)	7 (28.0%)	4 (36.4%)	5 (14.7%)	1 (16.7%)	2 (33.3%)	6 (30.0%)	6 (16.2%)
**[Dialysis]**									
I refer them to Hematology	2 (13.3%)	3 (17.6%)	3 (12.0%)	2 (18.2%)	4 (11.8%)	1 (16.7%)	1 (16.7%)	0 (0.0%)	8 (21.6%)
No	10 (66.7%)	13 (76.5%)	10 (40.0%)	4 (36.4%)	21 (61.8%)	5 (83.3%)	3 (50.0%)	11 (55.0%)	22 (59.5%)
Yes- All patients	0 (0.0%)	0 (0.0%)	4 (16.0%)	0 (0.0%)	4 (11.8%)	0 (0.0%)	0 (0.0%)	3 (15.0%)	1 (2.7%)
Yes-In some patients	3 (20.0%)	1 (5.9%)	8 (32.0%)	5 (45.5%)	5 (14.7%)	0 (0.0%)	2 (33.3%)	6 (30.0%)	6 (16.2%)
**[Kidney transplant]**									
I refer them to Hematology	0 (0.0%)	3 (17.6%)	3 (12.0%)	2 (18.2%)	3 (8.8%)	0 (0.0%)	1 (16.7%)	0 (0.0%)	6 (16.2%)
No	12 (80.0%)	13 (76.5%)	13 (52.0%)	5 (45.5%)	24 (70.6%)	5 (83.3%)	4 (66.7%)	15 (75.0%)	23 (62.2%)
Yes- All patients	0 (0.0%)	0 (0.0%)	1 (4.0%)	0 (0.0%)	1 (2.9%)	0 (0.0%)	0 (0.0%)	1 (5.0%)	0 (0.0%)
Yes-In some patients	3 (20.0%)	1 (5.9%)	8 (32.0%)	4 (36.4%)	6 (17.6%)	1 (16.7%)	1 (16.7%)	4 (20.0%)	8 (21.6%)

Table S15: monitoring DOAC levels in patietns with CKD (regardless of the ability of lab to perform such a test)

Variable	YEARS EXPERIANCE			PRACTICE SETTING				SPECIALITY	
	5-10 years	< 5 years	> 10 years	Both	Government Hospital	Outpatient dialysis units ONLY	Private Hospital	Hematology track	Nephrology track
Bleeding risk	7 (46.7%)	10 (58.8%)	22 (88.0%)	8 (72.7%)	21 (61.8%)	6 (100.0%)	4 (66.7%)	11 (55.0%)	28 (75.7%)

Degree of proteinuria	7 (46.7%)	10 (58.8%)	14 (56.0%)	5 (45.5%)	16 (47.1%)	4 (66.7%)	6 (100.0%)	8 (40.0%)	23 (62.2%)
I refer them to Nephrologist, Hematologist (or thromobosis expert), or Cardiologist	1 (6.7%)	4 (23.5%)	4 (16.0%)	3 (27.3%)	6 (17.6%)	0 (0.0%)	0 (0.0%)	5 (25.0%)	4 (10.8%)
I refer them to Nephrologist, or Hematologist (or thrombosis expert)	0 (0.0%)	0 (0.0%)	1 (4.0%)	1 (9.1%)	0 (0.0%)	0 (0.0%)	0 (0.0%)	0 (0.0%)	1 (2.7%)
Primary renal condition	6 (40.0%)	7 (41.2%)	9 (36.0%)	4 (36.4%)	9 (26.5%)	5 (83.3%)	4 (66.7%)	4 (20.0%)	18 (48.6%)
Serum albumin	12 (80.0%)	14 (82.4%)	16 (64.0%)	7 (63.6%)	24 (70.6%)	6 (100.0%)	5 (83.3%)	9 (45.0%)	33 (89.2%)

Table S16: factors that influence decisions about anticoagulation in nephrotic syndrome with normal kidney function (more than one option could be chosen)
